# Supplementary material for: Enabling programmable dynamic DNA chemistry using small-molecule DNA binders
Source: Nat Commun. 2023 Jul 17;14:4248. doi: 10.1038/s41467-023-40032-3 (PMC10352304; doi:10.1038/s41467-023-40032-3)
Supplement: Supplementary file 1 — Supplementary Information [file 41467_2023_40032_MOESM1_ESM.pdf]

Supplementary Information

Enabling programmable dynamic DNA chemistry using small-molecule DNA binders

Junpeng Xu[1,2], Guan Alex Wang[1,2], Lu Gao[1], Lang Wu,[1] Qian Lei[3], Hui Deng[3], Feng Li[1,2,*]

[1]Key Laboratory of Green Chemistry and Technology of Ministry of Education, College of Chemistry, Sicuan University, Chengdu, Sichuan, P. R. China, 610064

[2]Department of Chemistry, Centre for Biotechnology, Brock University, St. Catharines, Ontario, Canada, L2S 3A1

[3]Department of Respiratory and Critical Care Medicine, Institute of Respiratory Health, Targeted Tracer Research and Development Laboratory, West China Hospital, Sichuan University, Chengdu, Sichuan, P. R. China, 610064

[4]Med+X Center for Manufacturing, West China Hospital, Sichuan University, Chengdu, Sichuan, P. R. China, 610041

*Corresponding author: Feng Li, fli-scu@outlook.com

Table of Contents

Supplementary Methods **S4**

DNA oligonucleotides S4

DNA binders S4

Buffer conditions S5

Fluorescence Indicator Displacement (FID) Assay S5

Melting analysis of S20 .... S6

Molecular Docking S7

Cytotoxicity study (MTT assay) of S20 with three neoplastic cell lines S8

Theoretical Modeling and Fitting **S9**

Supplementary Figures **S10**

Schematic illustration of the workflow for performing BIND reaction S11

Kinetic and thermodynamic BIND profile for SG-I S11

Effect of duplex length on BIND S12

Schematic illustration of the workflow for investigating the kinetics of BIND S13

Kinetic curves for displacing CP duplex using varying concentrations of invader strand (I) in the presence of varying concentrations of SG S14

Observed rate constant kobs determined at varying concentrations of SG from 0 μM to 1.25 μM S14

Dissociative rate constant kdissociation and strand displacement rate constant kdisplacement were plotted as a function of binder (SYBR Green-I) concentration.. S15

Schematic illustration of the workflow for data fitting and simulationg against the theoretical model S16

Quantitative profiles of ΔG°_{BIND} as a function of increasing concentrations of binders S17

Evaluation of the potential influence of binder on fluorescence of Cy5 .... S18

Schematic illustration the workflow and mathematical transformation to determine the K_d and n of varying binders using BIND S19

A head-to-head comparation between the classic fluorescence turn-on assay (top) and BIND (bottom) for determining the K_d and binding site size of SG-I S20

BIND curves for representative small molecular DNA binders and non-binders....... S21

Schematic illustration the workflow of data fitting to key thermodynamic parameters S22

Profiling the binding thermodynamics of Actinomycin D using BIND S23

Profiling the binding thermodynamics of Berenil using BIND S24
Profiling the binding thermodynamics of DAPI using BIND..................................... S25
Profiling the binding thermodynamics of Daunorubicin using BIND S26
Profiling the binding thermodynamics of Doxorubicin using BIND.......................... S27
Profiling the binding thermodynamics of Echinomycin using BIND......................... S28
Profiling the binding thermodynamics of Ellipticine using BIND S29
Profiling the binding thermodynamics of Ethidium Bromide using BIND S30
Profiling the binding thermodynamics of Hoechst 33258 using BIND S31
Profiling the binding thermodynamics of Netropsin using BIND S32
Profiling the binding thermodynamics of Pico Green using BIND S33
Profiling the binding thermodynamics of Proflavine using BIND S34
Profiling the binding thermodynamics of Quinacrine using BIND S35
Profiling the binding thermodynamics of SG-I using BIND S36
Profiling the binding thermodynamics of Thiazole Orange using BIND S37
Determining sequence selectivity of DAPI S38
Determining sequence selectivity of Actinomycin D using GC and AT-rich sink probes S39
Determining sequence selectivity of Berenil using GC and AT-rich sink probes S40
Determining sequence selectivity of Doxorubicin using GC and AT-rich sink probes S41
Determining sequence selectivity of Ethidium Bromide using GC and AT-rich sink probes S42
Determining sequence selectivity of Hoechst 33258 using GC and AT-rich sink probes S43
Determining sequence selectivity of Netropsin using GC and AT-rich sink probes .. S44
Determining sequence selectivity of SG-I using GC and AT-rich sink probes S45
Determining sequence selectivity of Thiazole Orange using GC and AT-rich sink probes S46
Impact of GC content on BIND S47
Schematic illustration of the necessity of tandem BIND S48
Comparing BIND and FID for re-screening 15 known DNA binders S49
A list of eight positive hits achieved using tandem BIND HTS assay........................ S49
Determining sequence selectivity of newly discovered DNA binder S20 using GC and AT-rich sink probes.. S50

Profiling the binding thermodynamics of the newly discovered DNA binder S20 using BIND S51

Cytotoxicity study (MTT assay) of S20 with three neoplastic cell lines S52

BIND profiles for SG-I in the absence NaCl or the presence of carying Nacl concentrations........ S53

Supplementary Tables S54

DNA sequences and modifications S54

Comparison of dissociation constants and binding site sizes measured using BIND against reported values S55

Comparison of enthalpic contributions measured using BIND against reported values S56

Supplementary References S57

S1. Supplementary Methods

DNA Binders. Actinomycin D, Berenil, Daunorubicin, Doxorubicin, Echinomycin, Hoechst 33258, Ellipticine and Quinacrine were purchased from Cayman Chemical Company (Cayman Chemical, Ann Arbor, MI, United States). Crystal Violet,DAPI, DAPP, Ethidium Bromide (EtBr), Proflavine, $[Ru(phen)_3]Cl_2$, SYBR Green I (SG-I), Thiazole Orange and Thioflavin T were purchased from Sigma-Aldrich Chemical Company (Sigma-Aldrich, St. Louis, MO). Eva Green was purchased from Biotium, Inc (Biotium, Hayward, CA). Pico Green was purchased from Lumiprobe Corporation (Lumiprobe, Hunt Valley, MA). Netropsin was purchased from VWR International Company (VWR, Radnor, PA). Selleck's Express-Pick Library was provided by Professor Weimin Li at the Department of Respiratory and Critical Care Medicine, Targeted Tracer Research and Development Laboratory, West China hospital of Sichuan University.

Buffer conditions. DNA oligonucleotides were re-suspended by dissolving in deionized water and then stored at -20 °C. Unless indicated otherwise, 1 x PBS buffer (pH 7.4, purchased as 10 × PBS stock from Sigma) containing 1 mM $MgCl_2$ and 0.1% (v/v) TWEEN 20 was used to prepare the CP duplex. 1 × Tris EDTA (10 mM Tris-HCl, pH 8.0, 1 mM EDTA, purchased from Sigma as 100 × stock) buffer containing 0.1% (v/v) TWEEN 20 (Sigma) was used as the BIND reaction buffer.

Fluorescence Indicator Displacement (FID) Assay. Endpoint fluorescence measurement was used in FID assay for the high throughput screen (HTS) of new binders. Briefly, 20 nM CP was mixed 1 µM of EB in 1 x TE buffer and then incubated at 37 °C for 2 hours. To this reaction mixture, a candidate compound at final concentration of 30 µM was added. After another incubation at 37 °C for 2 hours, endpoint fluorescence was measured using BioTek Cytation 5 Multimode Microplate reader at excitation/emission wavelength at 522 nm/593 nm. The fluorescence signal in each well was normalized using a positive control containing 20 nM CP and 1 µM of EB and a negative control containing 20 nM CP in 1 x TE buffer.

Melting analysis of S20. Briefly, a mixture containing 200 nM CP and S20 in 1 x TE buffer containing 20 mM NaCl at final volume of 50 µL. The solution was then analyzed using Analytik Jena qTOWER3G quantitative PCR system by a temperature program including an initial incubation at 20 °C for 15 min and then increasing the temperature at a rate of 0.5 °C per minute until 90 °C. Fluorescence signal was recorded at a data acquisition rate of 1 data point per minute. Fluorescence excitation and emission wavelength was set at 640 nm/ 675 nm.

Molecular Docking. To visualize the binding mechanism between DNA and binders, specific binding models were obtained by downloading published crystal structures form Protein Data Band (https://www.rcsb.org/) or performing molecule docking. Specifically, the crystal structures of Actinomycin D, Berenil, DAPI, Echinomycin, Ellipticine, Hoechst 33258, Netropsin, Proflavine, and TOTO binding to DNA were downloaded from Protein Data Bank (https://www.rcsb.org/), and the binding modes were visualized by Pymol software (Table S1). While binding modes of Daunorubicin, Doxorubicin, Pico Green, Quinacrine, SYBR Green I, Thiazole Orange were simulated by molecular docking software Autodock 4.2. In detail, the .mol files of binders were obtained through Chemdraw software, and .pdb files of DNA were obtained through PDB. In the preliminary preparations for the molecular docking, the binders were successively hydrogenated, detected root, chosen torsion and finally converted into PDBQT files, while DNAs were similarly hydrogenated, removed water molecules, calculated gaseiger, assigned AD4 type, and finally converted to PDBQT files. The grid box was uniformly set to contain the entire DNA structure. During the docking process, the Lamarckian genetic algorithm was used for binder-DNA docking. Among them, the maximum number of energy evaluations was set to 2,500,000. The maximum number of generations was 27,000. The rate of gene mutation was 0.02. The rate of crossover was 0.8. Maximum number of top individuals that automatically survive was 1. Finally, we comprehensively evaluated and selected the optimal binding conformation of binder and DNA according to the stable binding energy, which were visualized with Pymol software.

Cytotoxicity study (MTT assay) of S20 with three neoplastic cell lines. Three cell lines Hela (ATCC, CCL-2), HepG2 (ATCC, HB-8065), and A549 (ATCC, CCL-185) were purchased from the American Type Culture Collection (ATCC, Manassas, VA). Cells were cultured in Dulbecco's modified Eagle medium containing 10% fetal bovine serum (Gibco), penicillin (100 U/mL), and streptomycin (100 μg/mL). Cells were seeded into 96-well plates (BIOFIL, catalog number TCP-011-096) at 1×10^4 cells per well and incubated for 12 h at 37 °C in 5% CO_2 to facilitate attachment. Cells were treated with different concentrations of compounds in DMEM with 10% FBS and then incubated for 24 h. Cells with no compounds added served as controls. After incubation, old media was removed, and cells were washed with PBS once before cell media was replaced with 120 μL of fresh media with MTT (0.5 mg/mL). Cells were incubated for another 1.5 h at 37 °C in 5% CO_2. Next, the media was replaced with 100 μL of DMSO and cell viability was determined by measuring the absorbance at 595 nm. Cell viability values were expressed as percentages and calculated as follows: Viability % = [Abs595 nm of treated sample) / [Abs595 nm of control] × 100%.

S2. Theoretical Modeling and Fitting

Thermodynamic Model

A theoretical model for BIND was established by considering the strand displacement to be an S_N1 reaction in the absence of the binder or the binder concentration was below the critical binder concentration (CBC). When the binder concentration was greater than CBC, the reaction was considered to go through an S_N2 strand displacement reaction pathway. To quantitatively profile BIND, we established a workflow for extracting critical thermodynamic parameters by combining experimental measurement and theoretical fitting (Fig. S2). We first derived the initial values of K_D and K_H in the absence of any binder by fitting the experimental data. Equilibrium constants when binder concentration equals to CBC were also determined. K_D values in the presence of binders with concentrations below CBC were then determined by fitting experimental data in a dissociation reaction of CP in the presence of binders but absence of the invader I (Fig. S1h). K_H and the overall strand displacement equilibriumK_{S_N1} could then be determined by fitting the strand displacement reaction using the S_N1 reaction mechanism and experimentally determined reaction yield in the presence of a given concentration of binder.

$$CP \rightarrow C + P,\ K_D \tag{1}$$

$$C + I \rightarrow CI,\ K_H \tag{2}$$

By further determining the Gibbs free energy of the reaction using the equilibrium constant (Fig. S2), we observed a linear relationship between ΔG_{S_N1} and the concentrations of DNA binders (Fig. S3). The similar mathematical treatment at the S_N2 region also revealed a linear relationship of ΔG_{S_N2} against binder concentration above CBC (Fig. S3).

$$CP + I \rightarrow CPI \rightarrow CI + P,\ K_{S_N2} \tag{3}$$

Kinetic Model

Kinetic model of BIND was established based on DNA strand-exchange model previously described by Reynaldo *et al*,[1] where both dissociative (S_N1) and sequential displacement (S_N2) pathways were considered for a toehold-free DNA strand exchange reaction. In this work, the observed rate constant k_{obs} was determined to be $k_{dissociative} + k_{displacement} \cdot [Invader]$, where $k_{dissociative}$ and $k_{displacement}$ were individual reaction rate constants for dissociative and sequential displacement pathways, respectively. Based on this model, we established the kinetic model for BIND, where the reaction rate

$$\frac{d[CP]}{dt} = -k_{dissociative} \cdot [CP] - k_{displacement} \cdot [CP][I]$$

$$\frac{d[CP]}{dt} = -\left(k_{dissociative} + k_{displacement}[I]\right)[CP]$$

If $[I]_0$ is in large excess:

$$\frac{d[CP]}{dt} \approx -k_{obs} \cdot [CP]$$

$$k_{obs} = k_{dissociative} + k_{displacement} \cdot [I]_0$$

therefore,

$$[CP] = [CP]_0 \cdot \exp\left(-k_{obs} \cdot t\right)$$

$$Yield_t = \exp\left(-k_{obs} \cdot t\right)$$

S3. Supplementary Figures

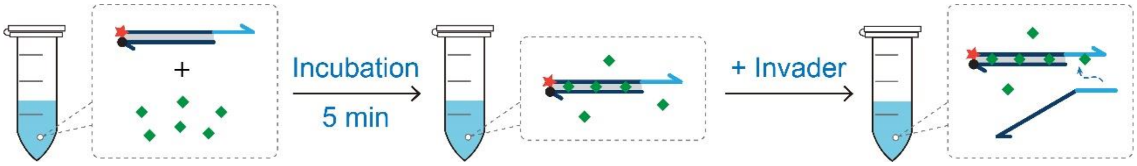

Supplementary Figure 1 | Schematic illustration of the workflow for performing BIND reaction.

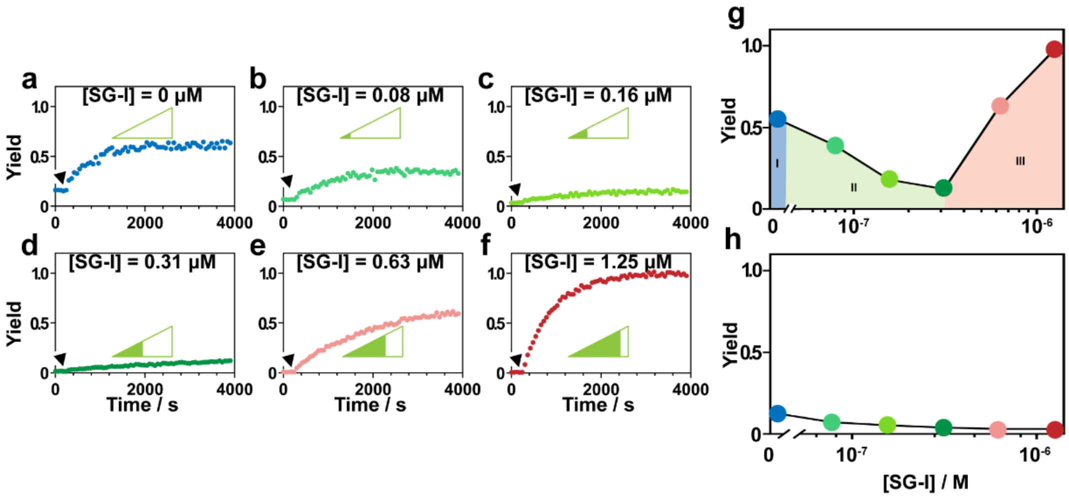

Supplementary Figure 2 | Kinetic and thermodynamic BIND profile for SG-I. (**a-f**) Representative kinetic profiles of strand displacement reactions in the absence (**a**) and increasing concentrations of SG-I (**b-f**). **g.** Thermodynamic profile of SG-I-mediated BIND reaction by plotting the strand displacement reaction yields at equilibrium against increasing concentrations of SG-I. **h.** Effect of increasing concentration of SG-I to the stability of CP duplex in the absence of the invader.

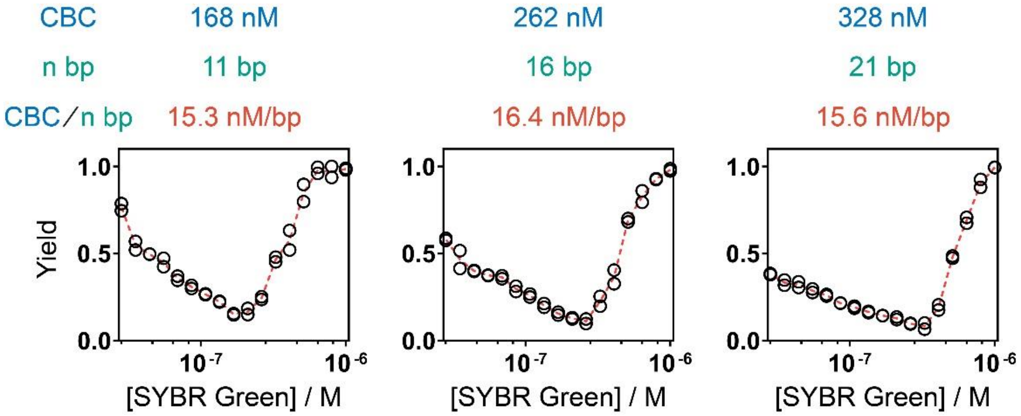

Supplementary Figure 3 | Effect of duplex length on BIND. BIND profiles were established for three duplex lengths (all with the same level of GC content), including 11 bp, 16 bp, and 21 bp. The initial fluorescence in the absence of binder was found to decrease as the length of duplex increased. This is to be expected as more complementary base pairs made CP duplex more stable even in the absence of metal cations. CBC was found to shift from a lower concentration of 168 nM to 328 nM when increasing the duplex length from 11 bp to 21 bp. Meanwhile, the ratio between CBC and duplex length was a constant.

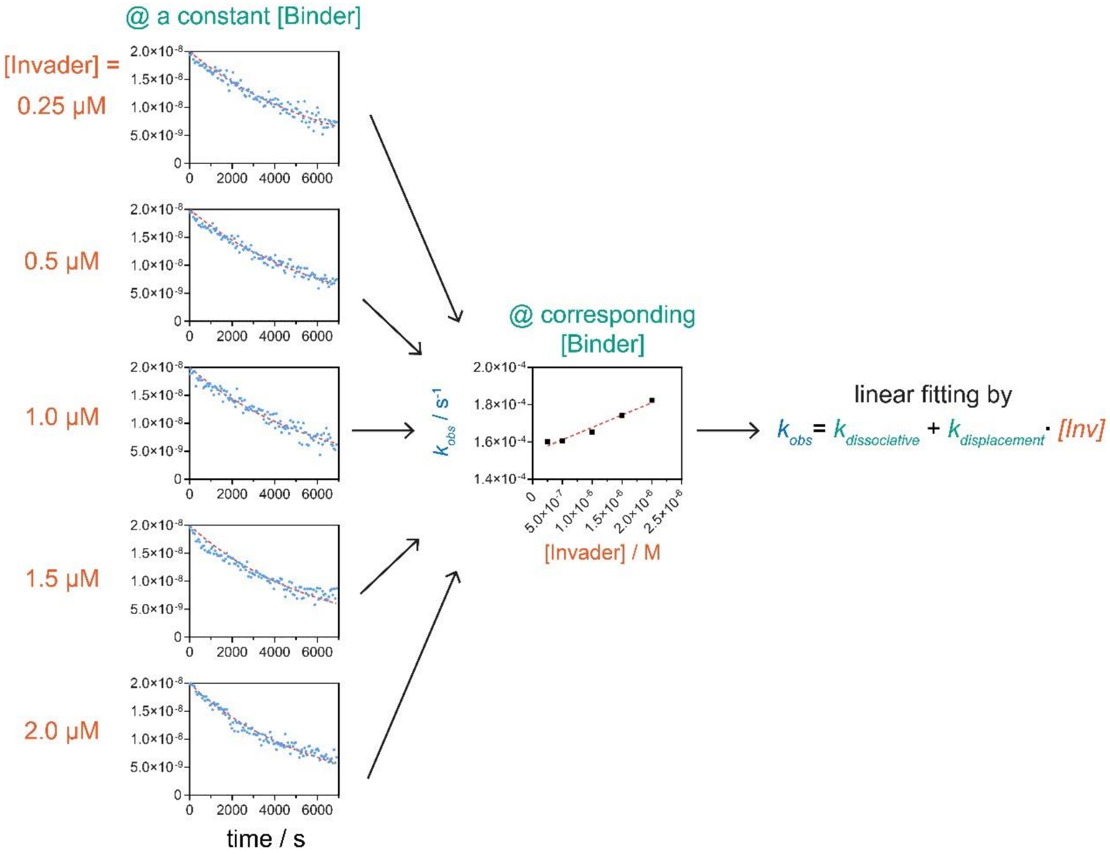

Supplementary Figure 4 | Schematic illustration of the workflow for investigating the kinetics of BIND. Kinetic curves were collected when invading CP duplex using increasing concentrations of invader I. The observed rate constant k_{obs} were determined at varying invader concentrations, which could then be used to determine $k_{dissociative}$ and $k_{displacement}$ through linear fitting. Briefly, at each invader concentration, the value of k_{obs} was fitted by the following function: $[CP]_t = [CP]_0 \cdot \exp{(-k_{obs} \cdot t)}$. A linear regression against the theoretical model was used to determine $k_{dissociation}$ and $k_{displacement}$ thereafter, $k_{obs} = k_{dissociative} + k_{displacement} \cdot [I]$. All fitting curves were shown as red lines.

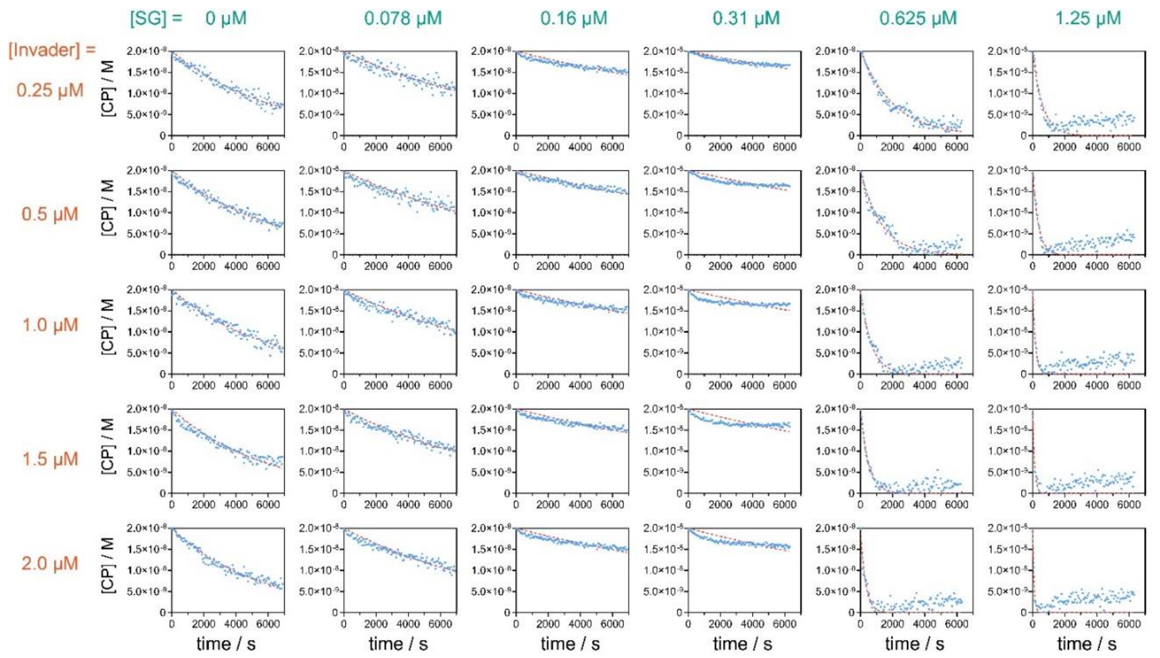

Supplementary Figure 5 | Kinetic curves for displacing CP duplex using varying concentrations of invader strand (I) in the presence of varying concentrations of SG. Values of k_{obs} were determined using the fitting approach as described in Fig. S4. All fitting curves were shown as red lines.

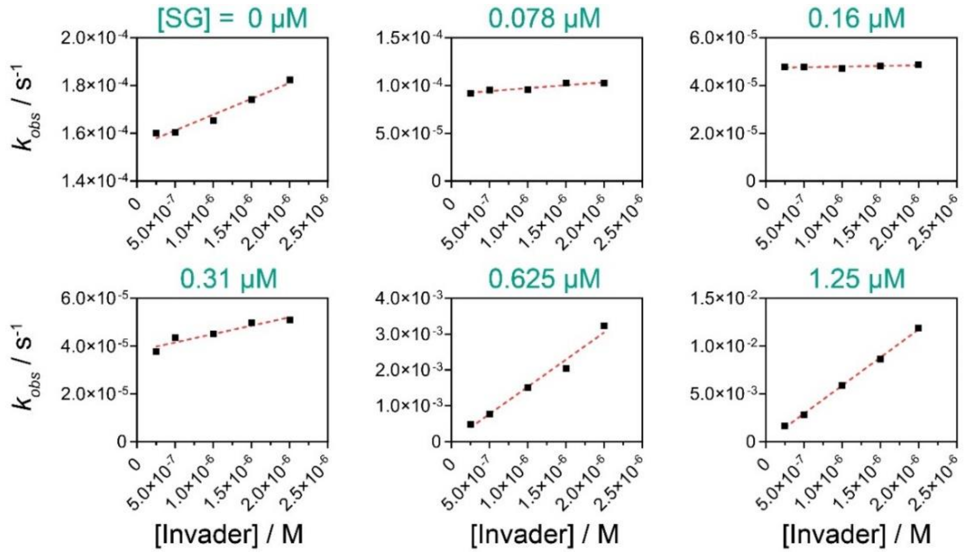

Supplementary Figure 6 | Observed rate constant k_{obs} determined at varying concentrations of SG from 0 μM to 1.25 μM. The linear regression fitting as described in Fig. S4 was then used to determine $k_{dissociative}$ and $k_{displacdment}$. All fitting curves were shown as red lines.

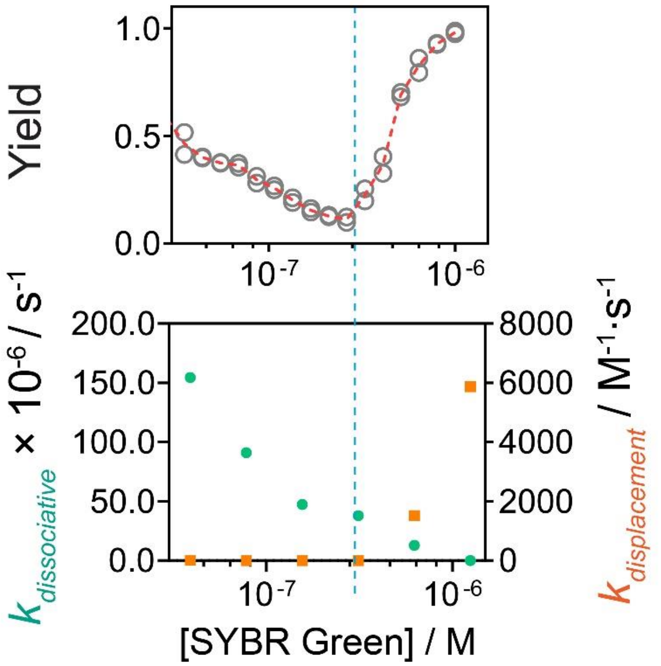

Supplementary Figure 7 | Dissociative rate constant $k_{dissociative}$ and strand displacement rate constant $k_{displacement}$ were plotted as a function of binder (SYBR Green-I) concentration. Changes in rate constants were highly consistent (bottom) with BIND profile, which confirmed the proposed reaction pathways at varying binder concentrations.

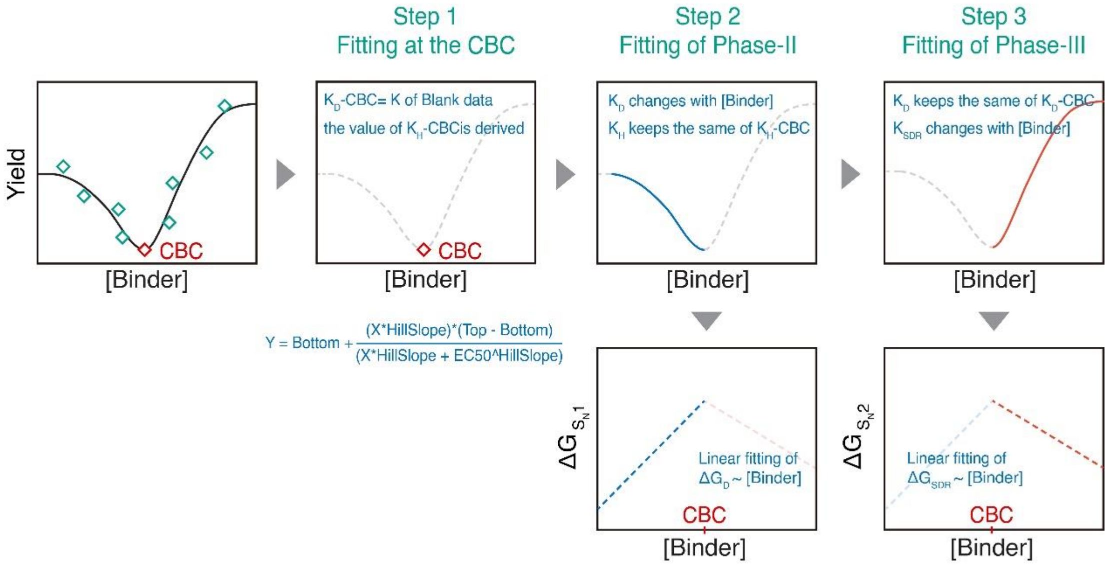

Supplementary Figure 8 | Schematic illustration of the workflow for data fitting and simulation against the theoretical model. A typical workflow involves the fitting at the critical binder concentration (CBC), at the binder concentration below CBC, and at the binder concentration above CBC. Binding constants and the reaction free energies were then determined through fitting and plotted against the binder concentrations.

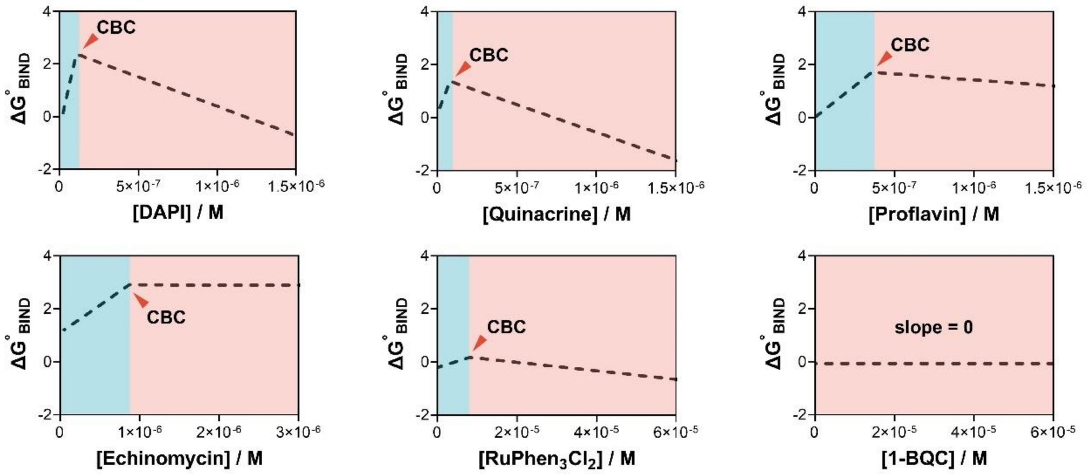

Supplementary Figure 9 | Quantitative profiles of ΔG°_{BIND} as a function of increasing concentrations of binders. Linear relationships were established for representative binders, including DAPI, Quinacrine, Proflavin, Echinomycin, and $RuPhen_3Cl_2$, as well as a positively charged non-binder, 1-BQC.

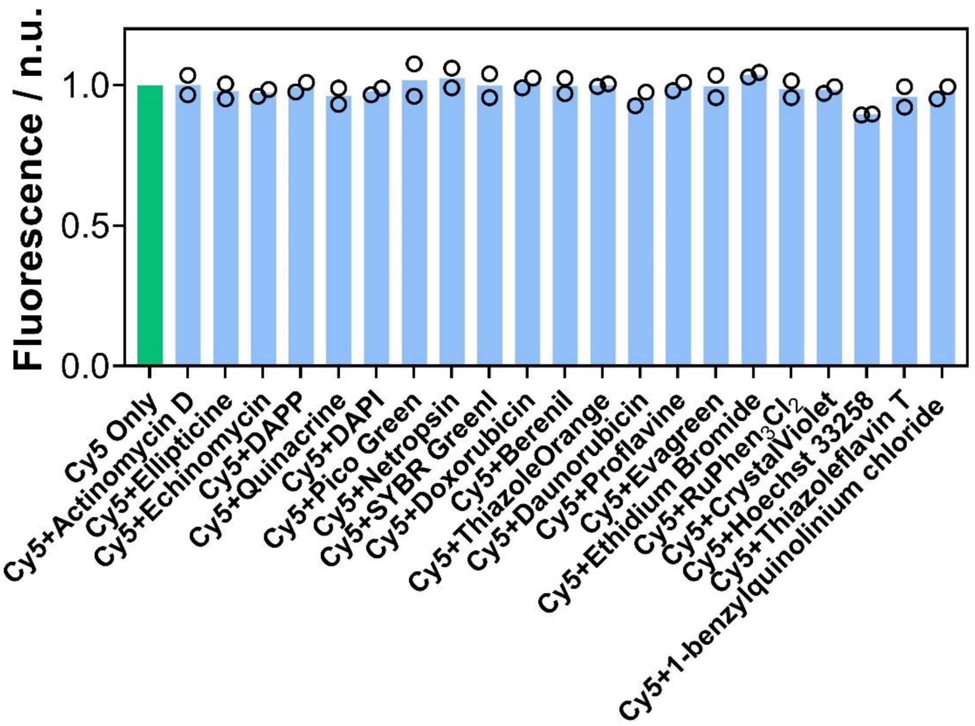

Supplementary Figure 10 | Evaluation of the potential influence of binder on fluorescence of Cy5. The Cy5-labeled CI duplex was fixed at 10 nM and binder concentrations were fixed at 5 μM. All fluorescence signals were normalized against 10 nM Cy5-labeled CI duplex in the absence of DNA binders. Technical replicates (n = 2) were used to ensure the reproducibility of the test.

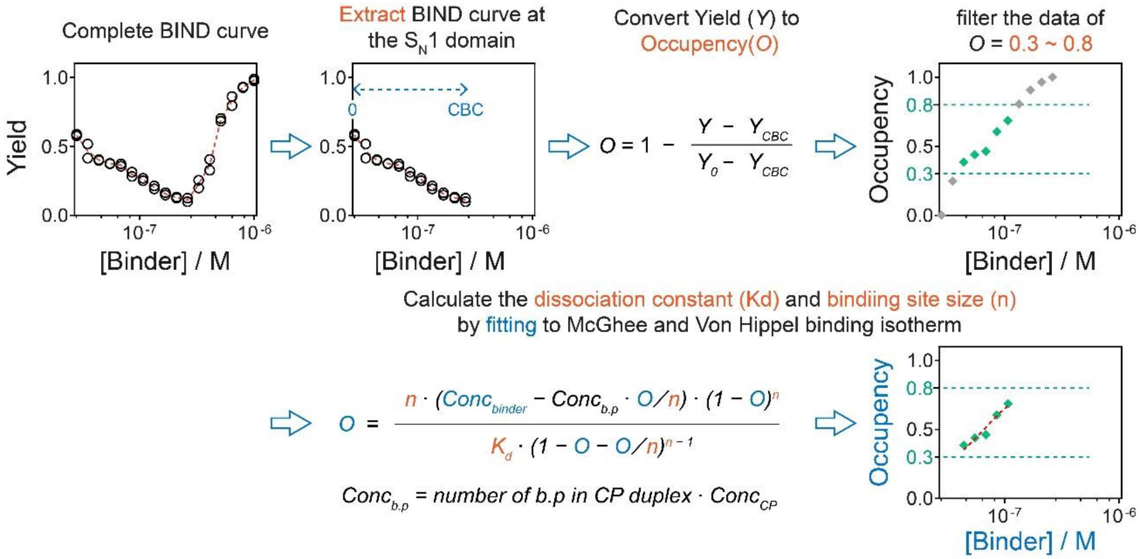

Supplementary Figure 11 | Schematic illustration the workflow and mathematical transformation to determine the K_d and n of varying binders using BIND. The fractional occupancy from 30% and 80% was determined to be the optimal range for determining critical binding parameters, including dissociation constants, and binding site sizes.[7]

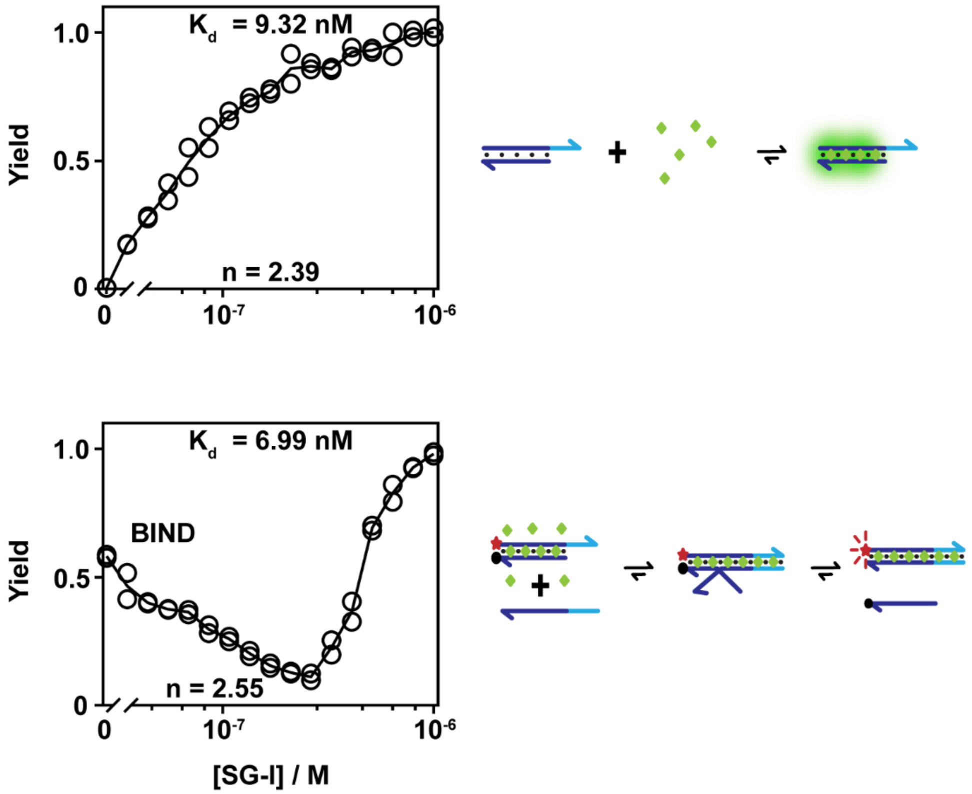

Supplementary Figure 12 | A head-to-head comparation between the classic fluorescence turn-on assay (top) and BIND (bottom) for determining the K_d and binding site size of SG-I. To ensure fair comparison, the same CP duplex with fixed concentration at 20 nM was used for both assays and highly consistent K_d and n values were determined in both assays.

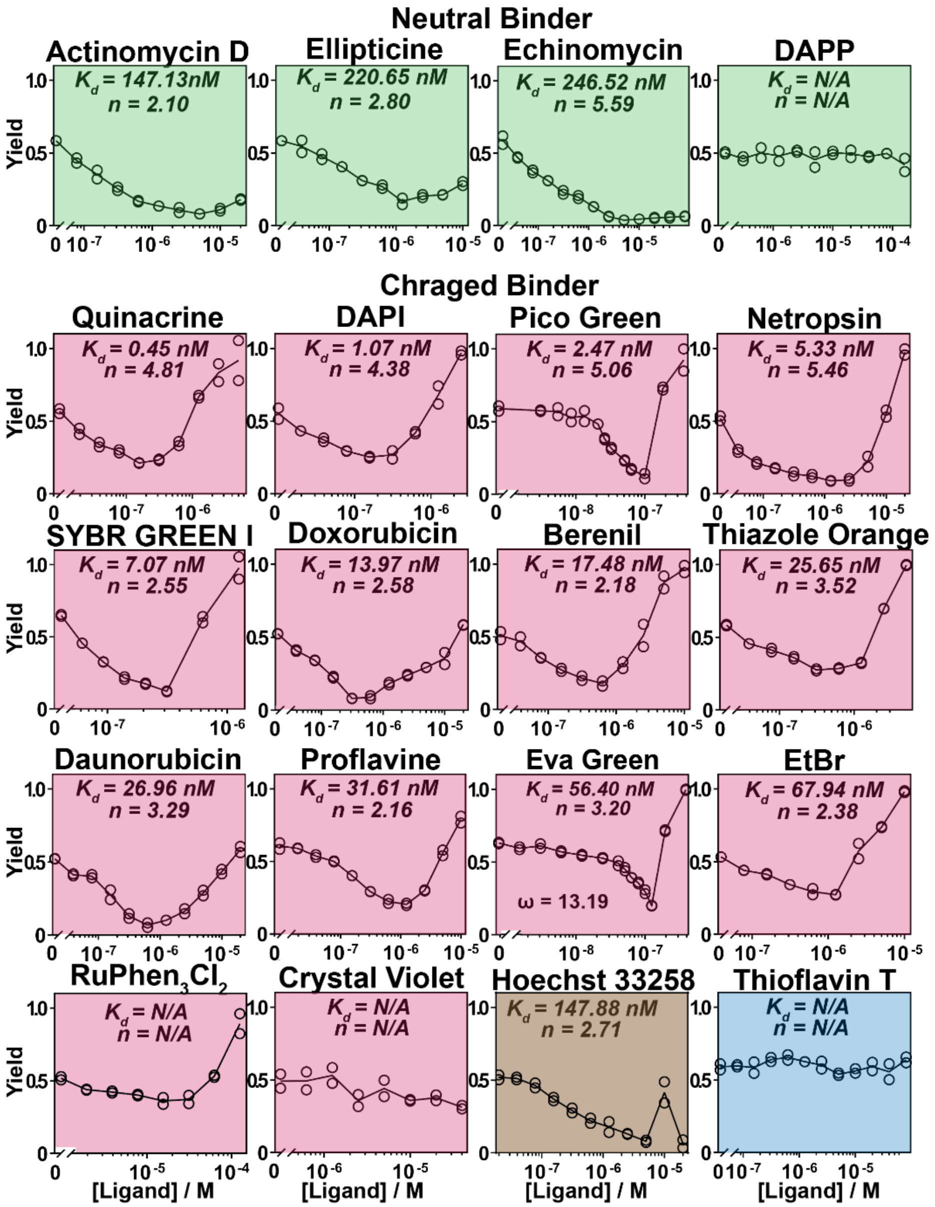

Supplementary Figure 13 | BIND curves for representative small molecular DNA binders and non-binders. Binding dissociation constants and binding site sizes were listed on the top of each BIND curve. Hoechst 33258 shows a sharp drop of fluorescence signal at high binder concentrations, which was a result of strong inner filter effect for the high concentration of Hoechst 33258 dye.

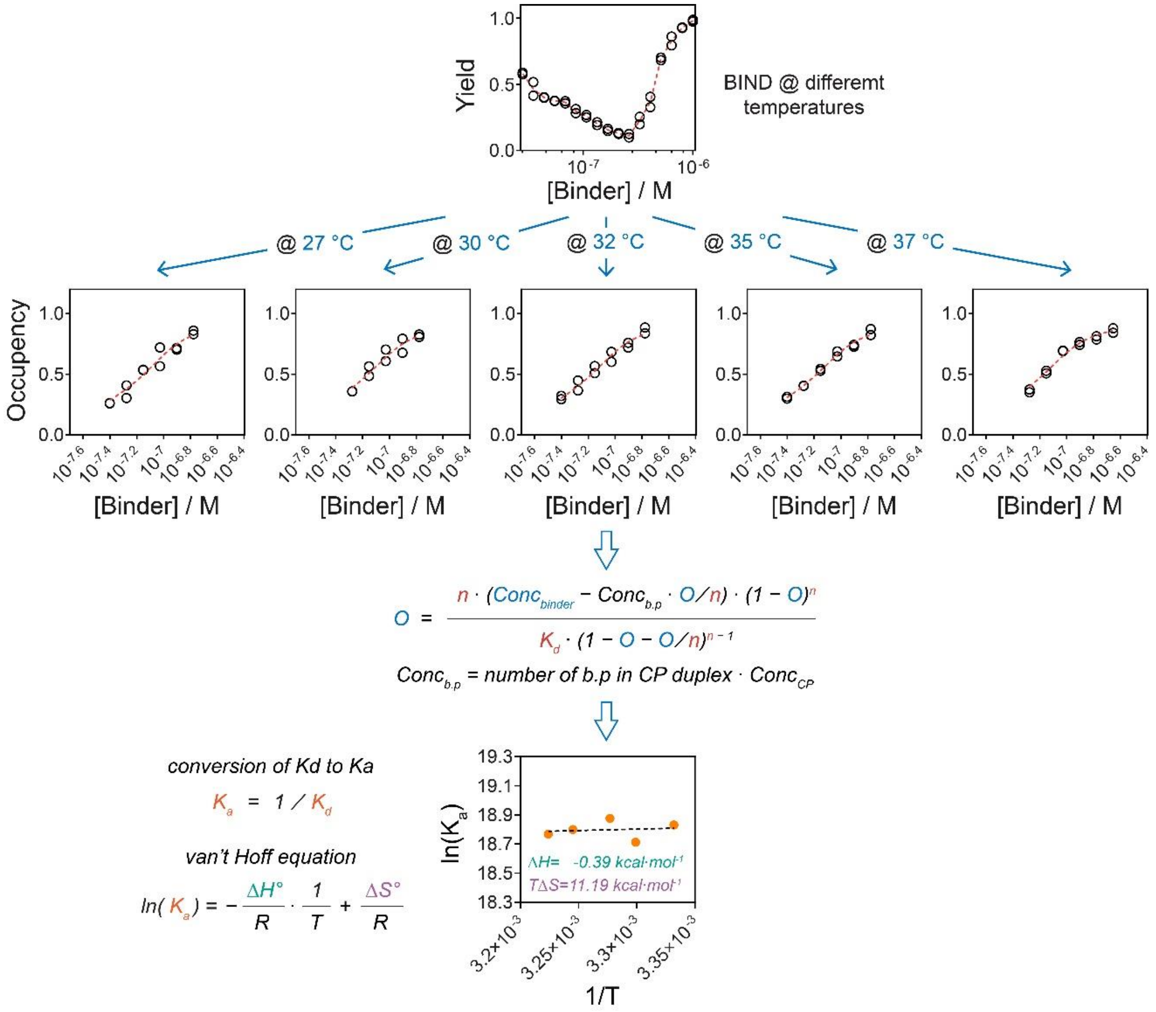

Supplementary Figure 14 | Schematic illustration the workflow of data fitting to key thermodynamic parameters. A typical workflow involves the collection of BIND curves and binding constants at varying temperatures and then the fitting using Van't Hoff equation to determine ΔH and ΔS.

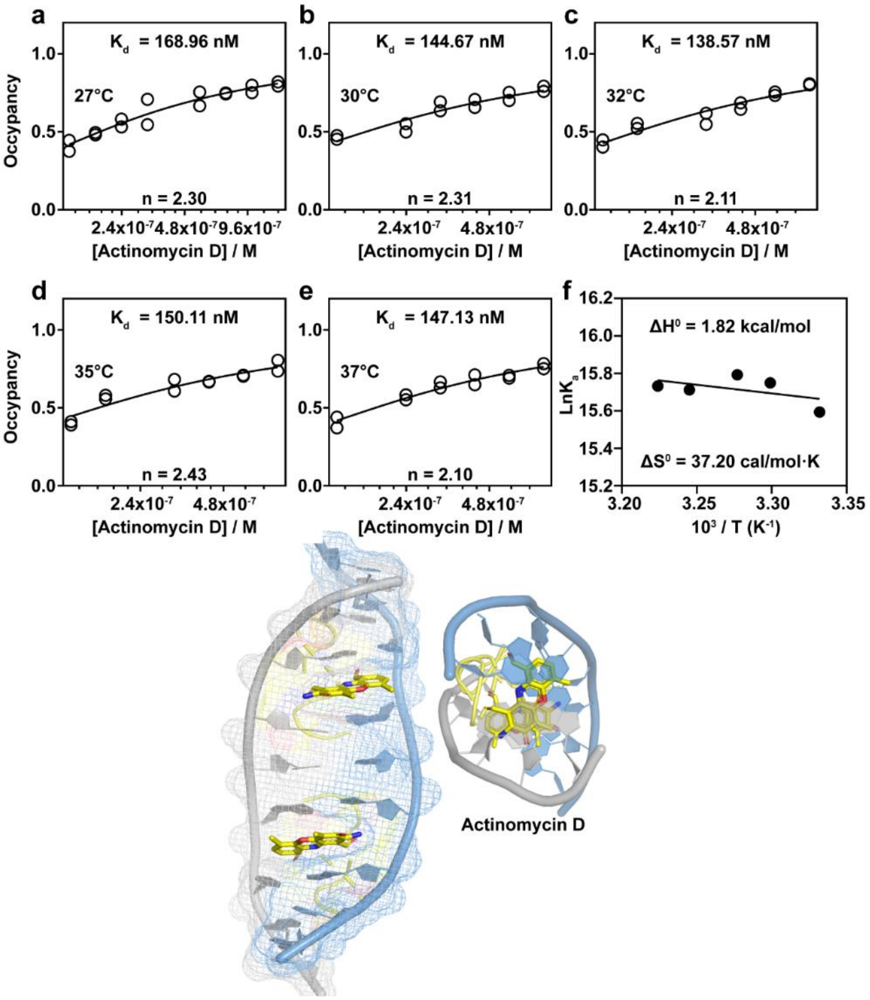

Supplementary Figure 15 | Profiling the binding thermodynamics of Actinomycin D using BIND. (a-e) BIND-based determination of dissociation constants at 27 ºC (**a**), 30 ºC (**b**), 32 ºC (**c**), 35 ºC (**d**) and 37 ºC (**e**). **(f)** Determination of △H and △S using linear fitting against Van't Hoff equation. Binding curves established using the S_N1 region of BIND was used to determine the K_d at varying temperatures. Van't Hoff plot was then established to determine the ΔHº and ΔSº. The binding mode was further visualized by molecular docking as outlined in the supplementary experimental section. The DNA 3D image was extracted from PDB file 1MNV.

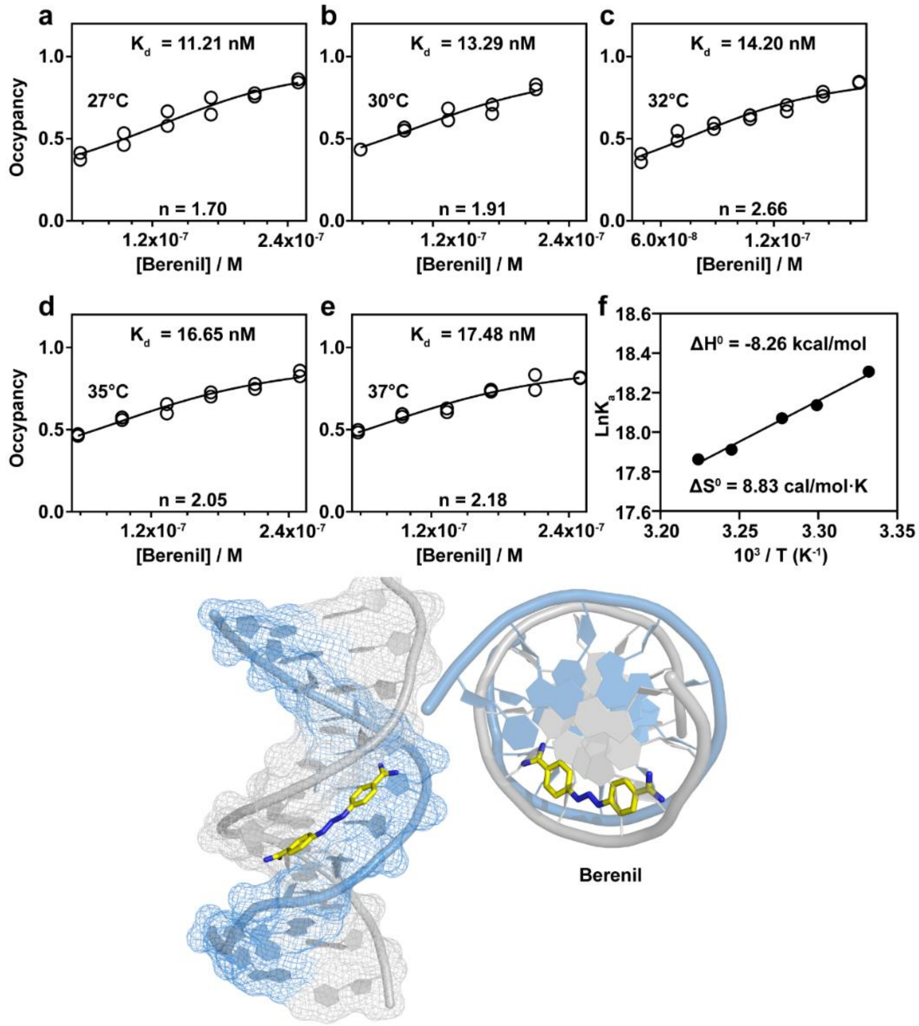

Supplementary Figure 16 | Profiling the binding thermodynamics of Berenil using BIND. (a-e) BIND-based determination of dissociation constants at 27 ºC (**a**), 30 ºC (**b**), 32 ºC (**c**), 35 ºC (**d**) and 37 ºC (**e**). **(f)** Determination of △H and △S using linear fitting against Van't Hoff equation. Binding curves established using the S_N1 region of BIND was used to determine the K_d at varying temperatures. Van't Hoff plot was then established to determine the ΔH^o and ΔS^o. The binding mode was further visualized by molecular docking as outlined in the supplementary experimental section. The DNA 3D image was extracted from PDB file 2GVR.

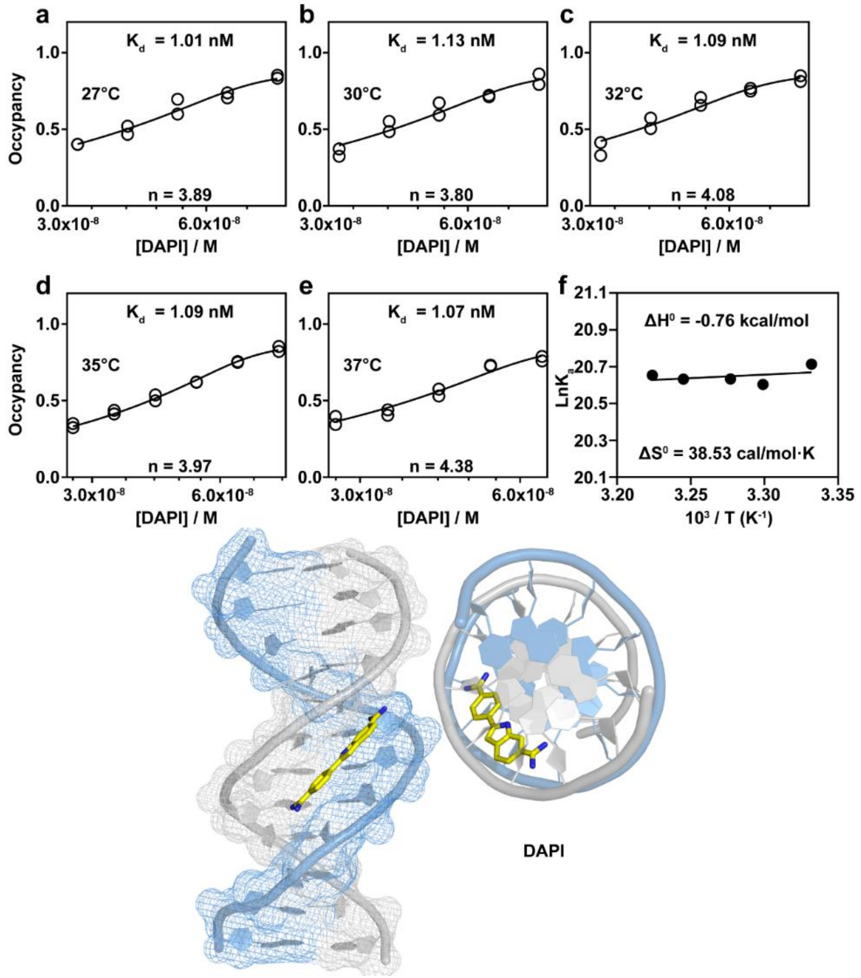

Supplementary Figure 17 | Profiling the binding thermodynamics of DAPI using BIND. (a-e) BIND-based determination of dissociation constants at 27 ºC (**a**), 30 ºC (**b**), 32 ºC (**c**), 35 ºC (**d**) and 37 ºC (**e**). **(f)** Determination of △H and △S using linear fitting against Van't Hoff equation. Binding curves established using the S_N1 region of BIND was used to determine the K_d at varying temperatures. Van't Hoff plot was then established to determine the ΔHº and ΔSº. The binding mode was further visualized by molecular docking as outlined in the supplementary experimental section. The DNA 3D image was extracted from PDB file 1D30.

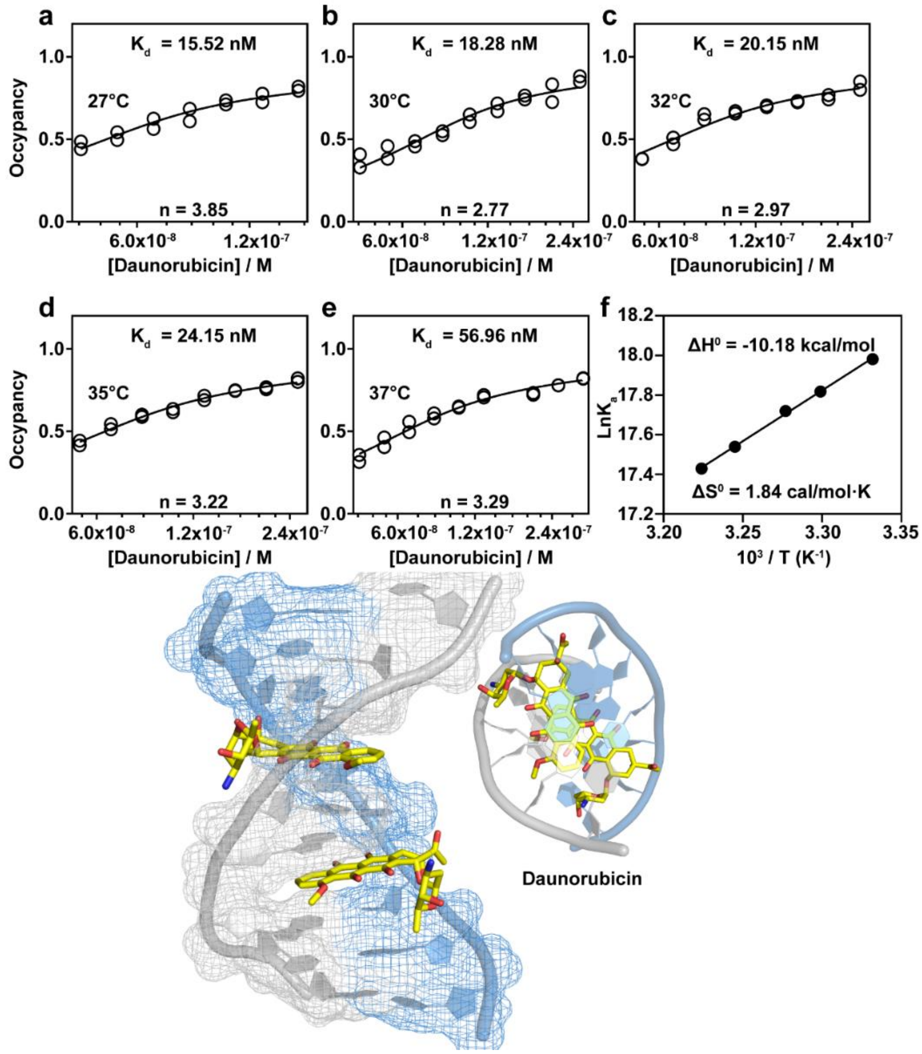

Supplementary Figure 18 | Profiling the binding thermodynamics of Daunorubicin using BIND. (a-e) BIND-based determination of dissociation constants at 27 ºC (**a**), 30 ºC (**b**), 32 ºC (**c**), 35 ºC (**d**) and 37 ºC (**e**). **(f)** Determination of △H and △S using linear fitting against Van't Hoff equation. Binding curves established using the S_N1 region of BIND was used to determine the K_d at varying temperatures. Van't Hoff plot was then established to determine the ΔH^o and ΔS^o. The binding mode was further visualized by molecular docking as outlined in the supplementary experimental section. The DNA 3D image was extracted from PDB file 108D.

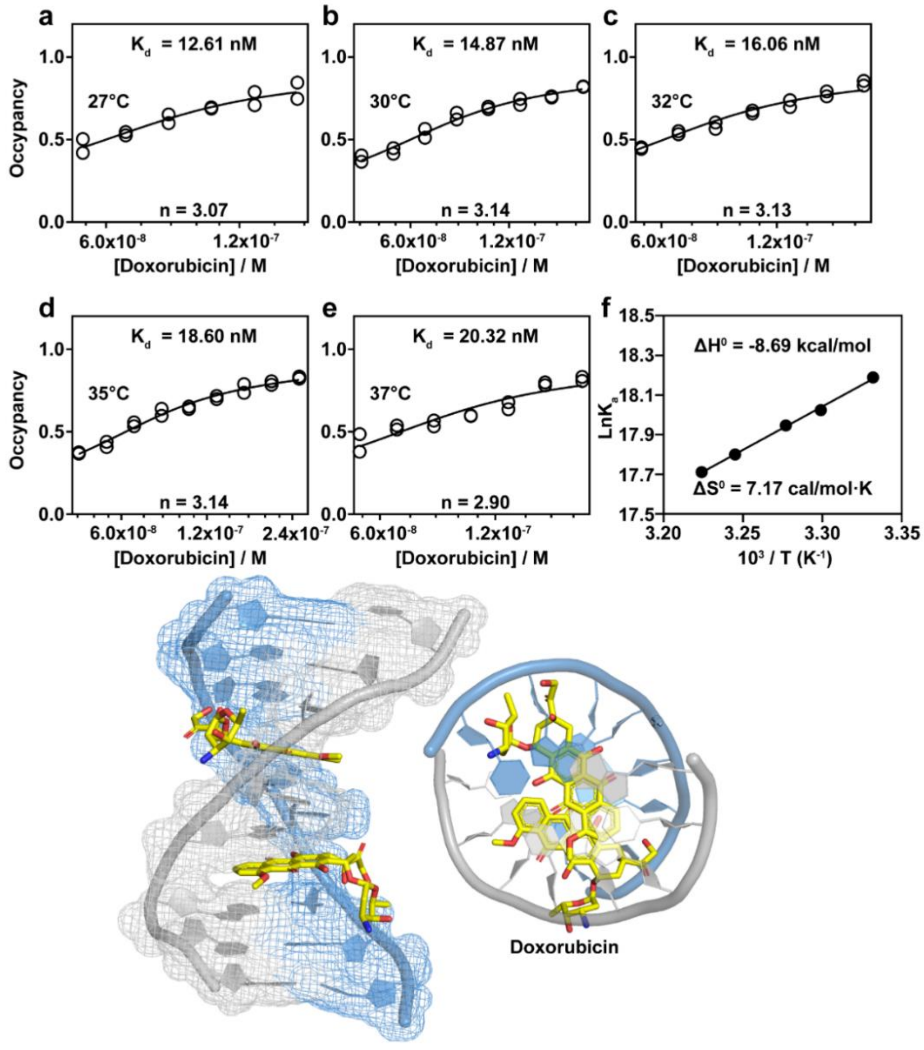

Supplementary Figure 19 | Profiling the binding thermodynamics of Doxorubicin using BIND. (a-e) BIND-based determination of dissociation constants at 27 ºC (**a**), 30 ºC (**b**), 32 ºC (**c**), 35 ºC (**d**) and 37 ºC (**e**). **(f)** Determination of △H and △S using linear fitting against Van't Hoff equation. Binding curves established using the S_N1 region of BIND was used to determine the K_d at varying temperatures. Van't Hoff plot was then established to determine the ΔH^o and ΔS^o. The binding mode was further visualized by molecular docking as outlined in the supplementary experimental section. The DNA 3D image was extracted from PDB file 108D.

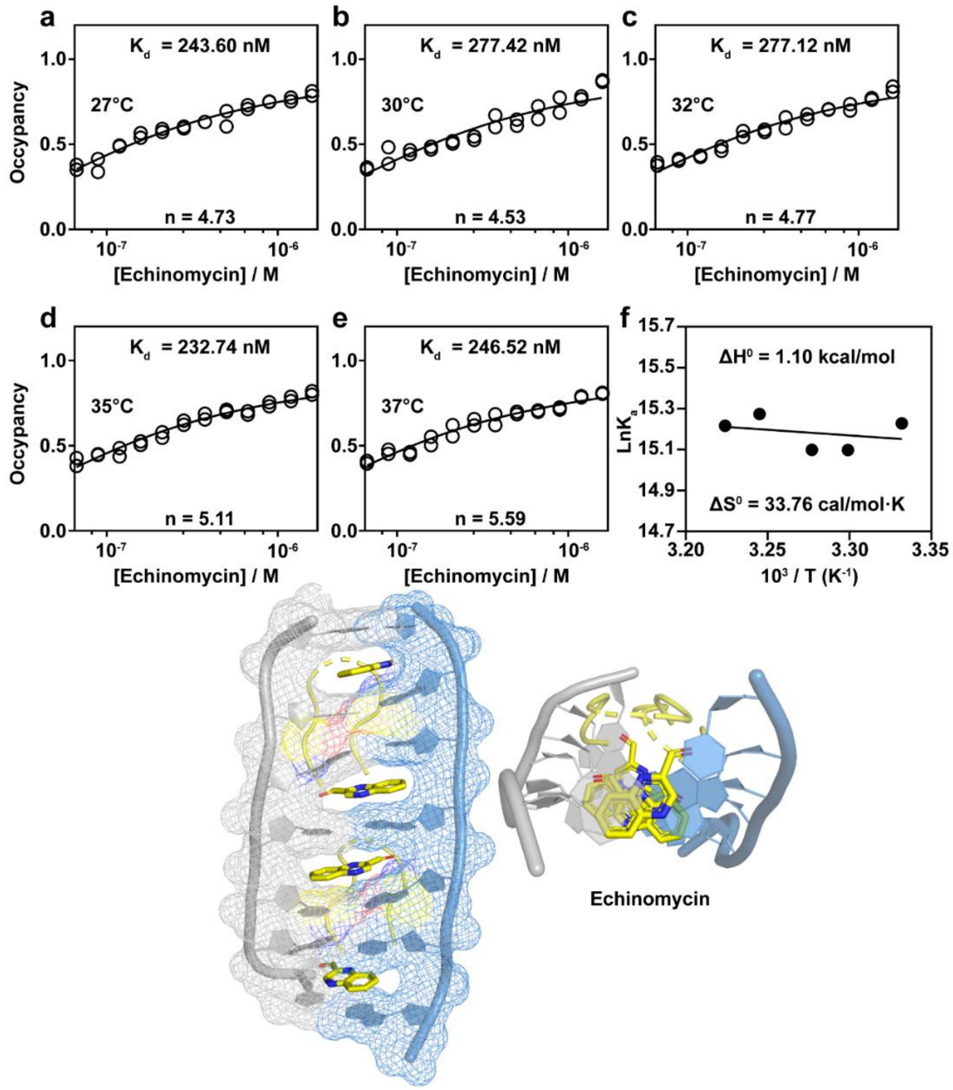

Supplementary Figure 20 | Profiling the binding thermodynamics of Echinomycin using BIND. (a-e) BIND-based determination of dissociation constants at 27 ºC (**a**), 30 ºC (**b**), 32 ºC (**c**), 35 ºC (**d**) and 37 ºC (**e**). **(f)** Determination of △H and △S using linear fitting against Van't Hoff equation. Binding curves established using the S_N1 region of BIND was used to determine the K_d at varying temperatures. Van't Hoff plot was then established to determine the ΔHº and ΔSº. The binding mode was further visualized by molecular docking as outlined in the supplementary experimental section. The DNA 3D image was extracted from PDB file 5YTY.

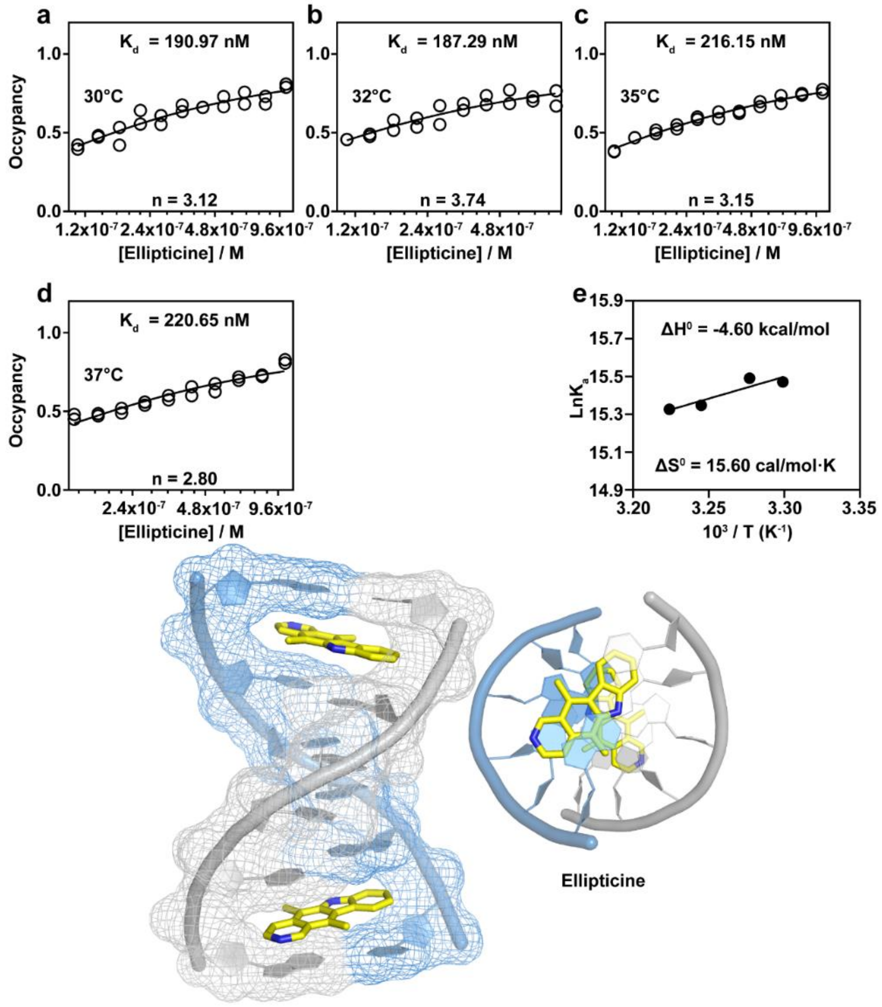

Supplementary Figure 21 | Profiling the binding thermodynamics of Ellipticine using BIND. (a-d) BIND-based determination of dissociation constants at 30 ºC (**a**), 32 ºC (**b**), 35 ºC (**c**), and 37 ºC (**d**). **(e)** Determination of △H and △S using linear fitting against Van't Hoff equation. Binding curves established using the S_N1 region of BIND was used to determine the K_d at varying temperatures. Van't Hoff plot was then established to determine the ΔHº and ΔSº. The binding mode was further visualized by molecular docking as outlined in the supplementary experimental section. The DNA 3D image was extracted from PDB file 1Z3F.

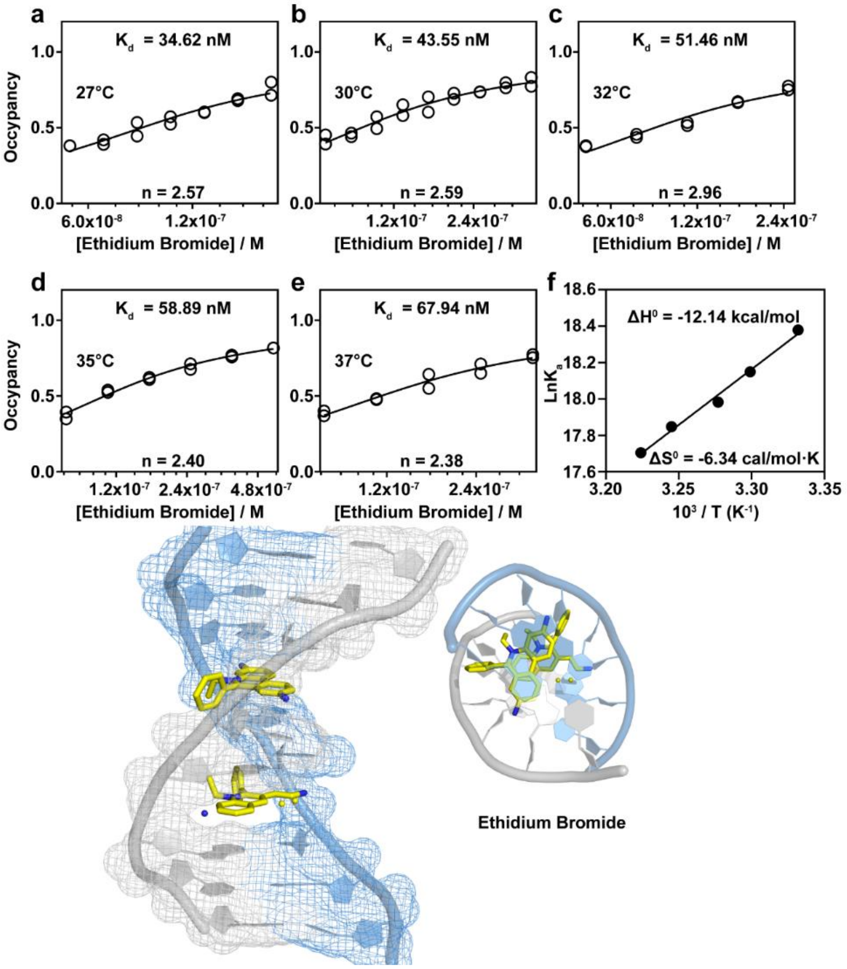

Supplementary Figure S22 | Profiling the binding thermodynamics of Ethidium Bromide using BIND. (a-e) BIND-based determination of dissociation constants at 27 ºC (**a**), 30 ºC (**b**), 32 ºC (**c**), 35 ºC (**d**) and 37 ºC (**e**). **(f)** Determination of △H and △S using linear fitting against Van't Hoff equation. Binding curves established using the S_N1 region of BIND was used to determine the K_d at varying temperatures. Van't Hoff plot was then established to determine the ΔHº and ΔSº. The binding mode was further visualized by molecular docking as outlined in the supplementary experimental section. The DNA 3D image was extracted from PDB file 108D.

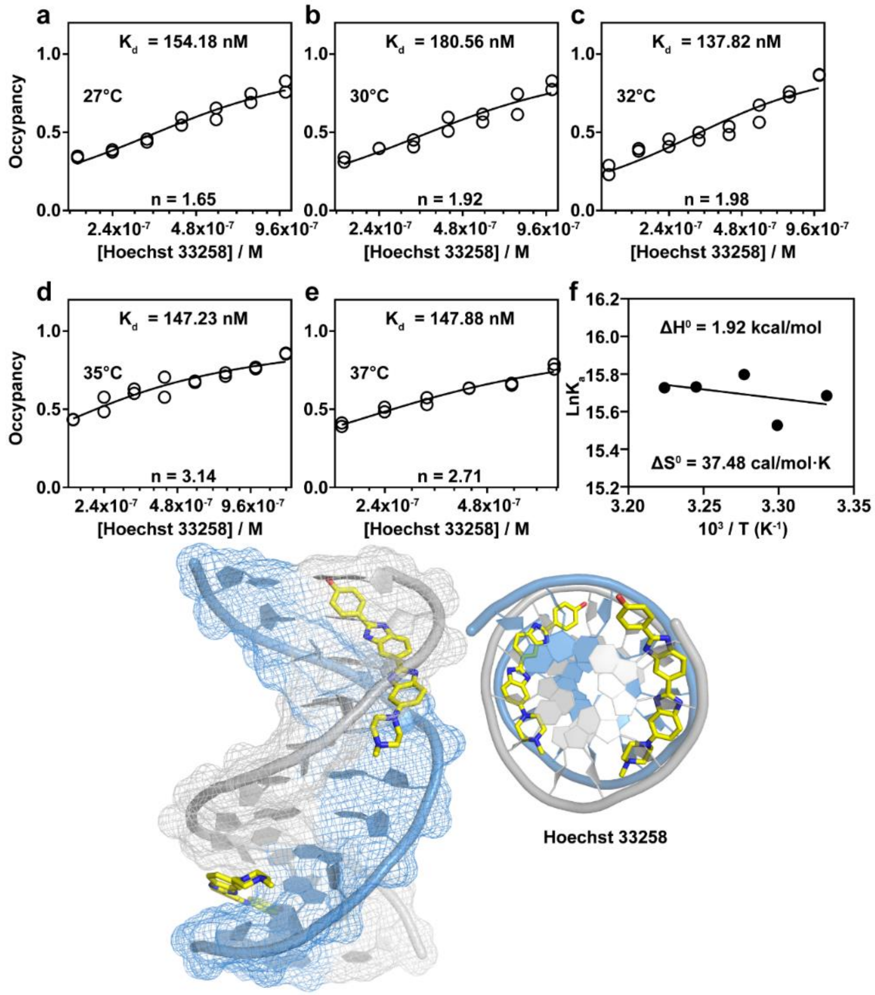

Supplementary Figure 23 | Profiling the binding thermodynamics of Hoechst 33258 using BIND. (a-e) BIND-based determination of dissociation constants at 27 ºC (**a**), 30 ºC (**b**), 32 ºC (**c**), 35 ºC (**d**) and 37 ºC (**e**). **(f)** Determination of △H and △S using linear fitting against Van't Hoff equation. Binding curves established using the S_N1 region of BIND was used to determine the K_d at varying temperatures. Van't Hoff plot was then established to determine the ΔHº and ΔSº. The binding mode was further visualized by molecular docking as outlined in the supplementary experimental section. The DNA 3D image was extracted from PDB file 1QSX.

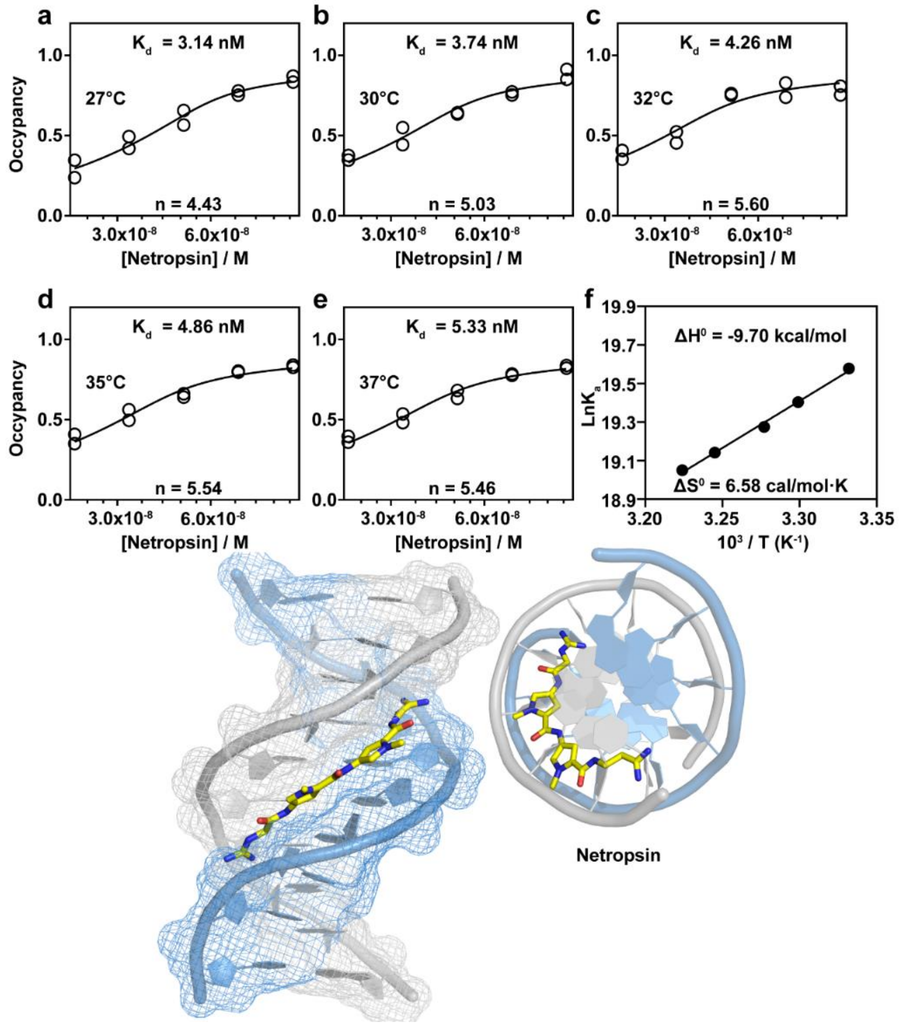

Supplementary Figure 24 | Profiling the binding thermodynamics of Netropsin using BIND. (a-e) BIND-based determination of dissociation constants at 27 ºC (**a**), 30 ºC (**b**), 32 ºC (**c**), 35 ºC (**d**) and 37 ºC (**e**). **(f)** Determination of △H and △S using linear fitting against Van't Hoff equation. Binding curves established using the S_N1 region of BIND was used to determine the K_d at varying temperatures. Van't Hoff plot was then established to determine the ΔHº and ΔSº. The binding mode was further visualized by molecular docking as outlined in the supplementary experimental section. The DNA 3D image was extracted from PDB file 2LWH.

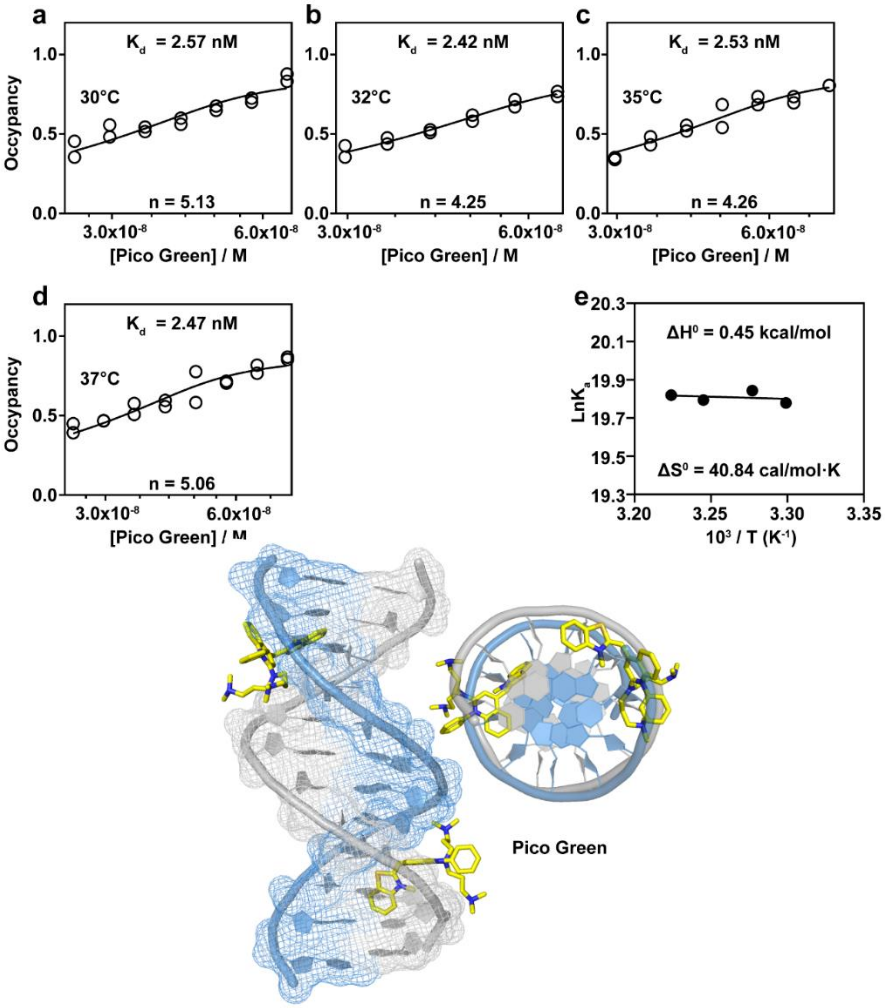

Supplementary Figure 25 | Profiling the binding thermodynamics of Pico Green using BIND. (a-d) BIND-based determination of dissociation constants at 30 ºC (**a**), 32 ºC (**b**), 35 ºC (**c**) and 37 ºC (**d**). **(e)** Determination of △H and △S using linear fitting against Van't Hoff equation. Binding curves established using the S_N1 region of BIND was used to determine the K_d at varying temperatures. Van't Hoff plot was then established to determine the ΔHº and ΔSº. The binding mode was further visualized by molecular docking as outlined in the supplementary experimental section. The DNA 3D image was extracted from PDB file 1D30.

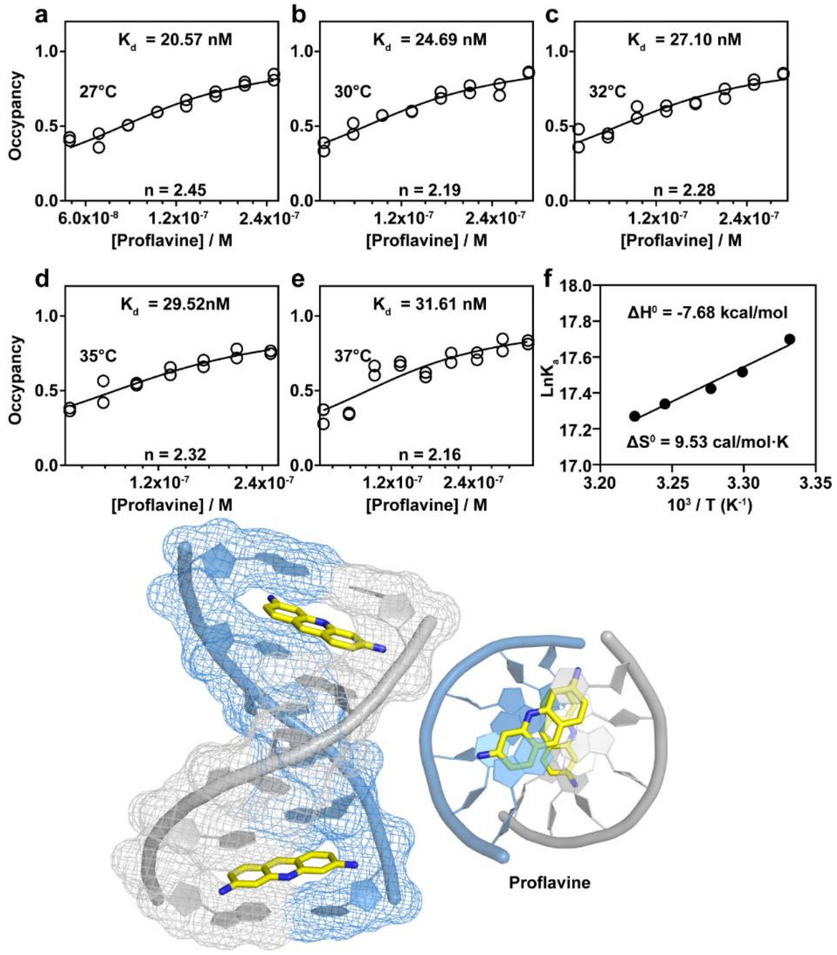

Supplementary Figure 26 | Profiling the binding thermodynamics of Proflavine using BIND. (a-e) BIND-based determination of dissociation constants at 27 ºC (**a**), 30 ºC (**b**), 32 ºC (**c**), 35 ºC (**d**) and 37 ºC (**e**). **(f)** Determination of △H and △S using linear fitting against Van't Hoff equation. Binding curves established using the S_N1 region of BIND was used to determine the K_d at varying temperatures. Van't Hoff plot was then established to determine the ΔHº and ΔSº. The binding mode was further visualized by molecular docking as outlined in the supplementary experimental section. The DNA 3D image was extracted from PDB file 3FT6.

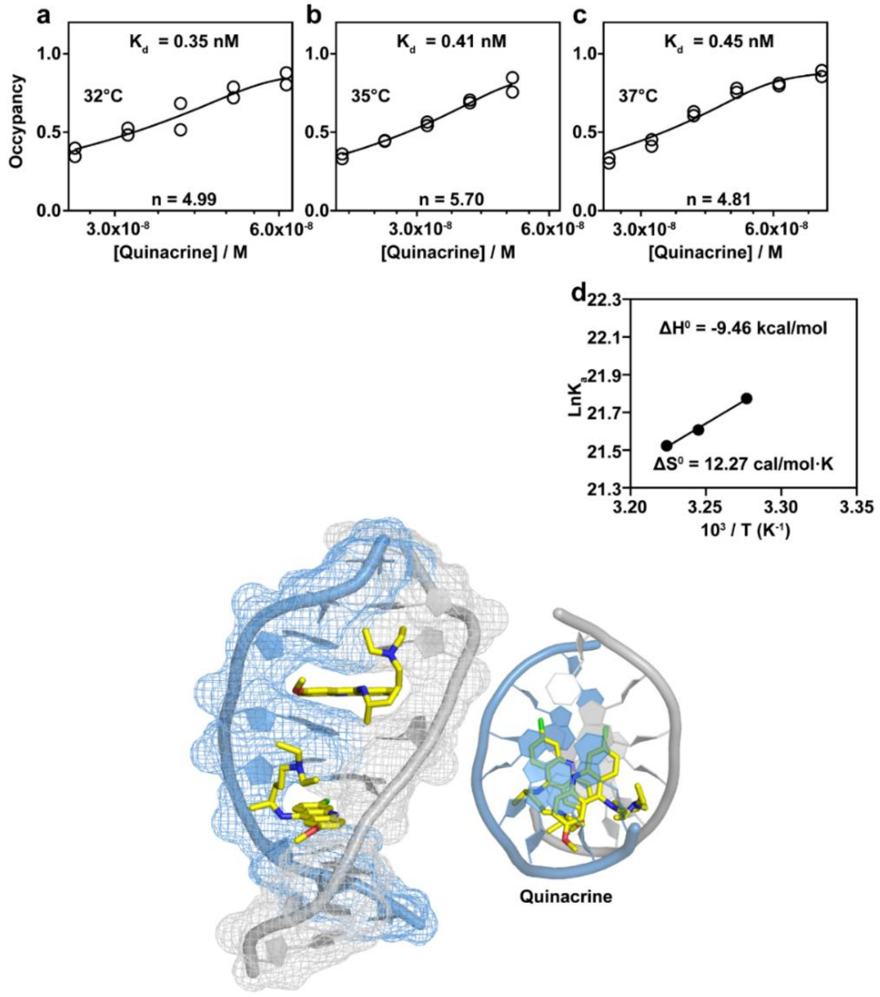

Supplementary Figure 27 | Profiling the binding thermodynamics of Quinacrine using BIND. (a-c) BIND-based determination of dissociation constants at 32 ºC (**a**), 35 ºC (**b**) and 37 ºC (**c**). **(d)** Determination of △H and △S using linear fitting against Van't Hoff equation. Binding curves established using the S_N1 region of BIND was used to determine the K_d at varying temperatures. Van't Hoff plot was then established to determine the ΔH^o and ΔS^o. The binding mode was further visualized by molecular docking as outlined in the supplementary experimental section. The DNA 3D image was extracted from PDB file 108D.

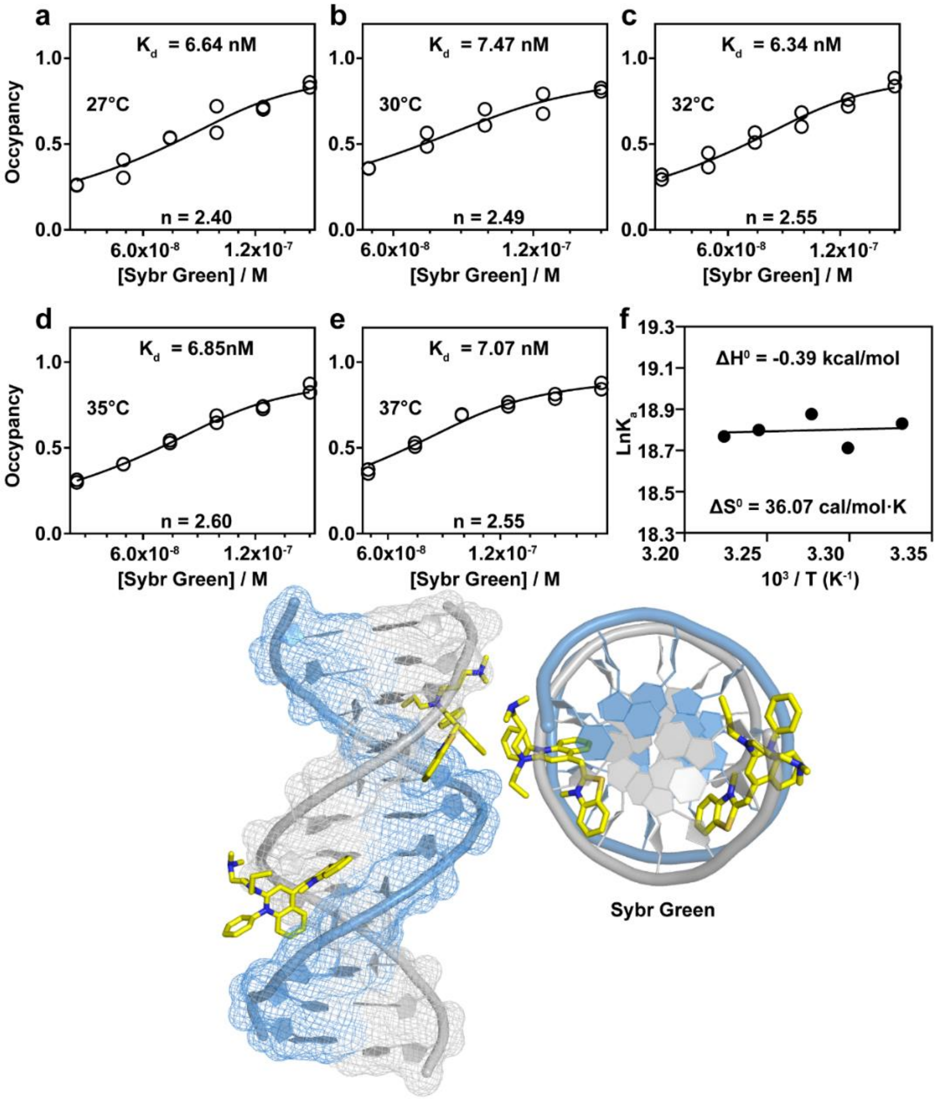

Supplementary Figure 28 | Profiling the binding thermodynamics of SG-I using BIND. **(a-e)** BIND-based determination of dissociation constants at 27 ºC (**a**), 30 ºC (**b**), 32 ºC (**c**), 35 ºC (**d**) and 37 ºC (**e**). **(f)** Determination of △H and △S using linear fitting against Van't Hoff equation. Binding curves established using the S_N1 region of BIND was used to determine the K_d at varying temperatures. Van't Hoff plot was then established to determine the ΔHº and ΔSº. The binding mode was further visualized by molecular docking as outlined in the supplementary experimental section. The DNA 3D image was extracted from PDB file 1D30.

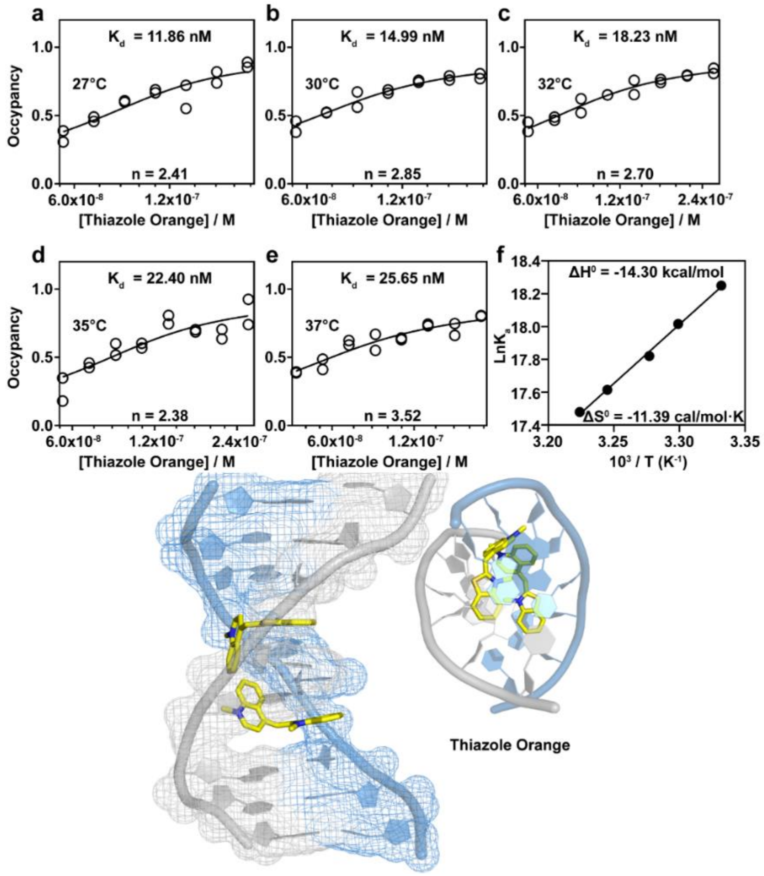

Supplementary Figure 29 | Profiling the binding thermodynamics of Thiazole Orange using BIND. (a-e) BIND-based determination of dissociation constants at 27 ºC (**a**), 30 ºC (**b**), 32 ºC (**c**), 35 ºC (**d**) and 37 ºC (**e**). **(f)** Determination of △H and △S using linear fitting against Van't Hoff equation. Binding curves established using the S_N1 region of BIND was used to determine the K_d at varying temperatures. Van't Hoff plot was then established to determine the ΔH° and ΔS°. The binding mode was further visualized by molecular docking as outlined in the supplementary experimental section. The DNA 3D image was extracted from PDB file 108D.

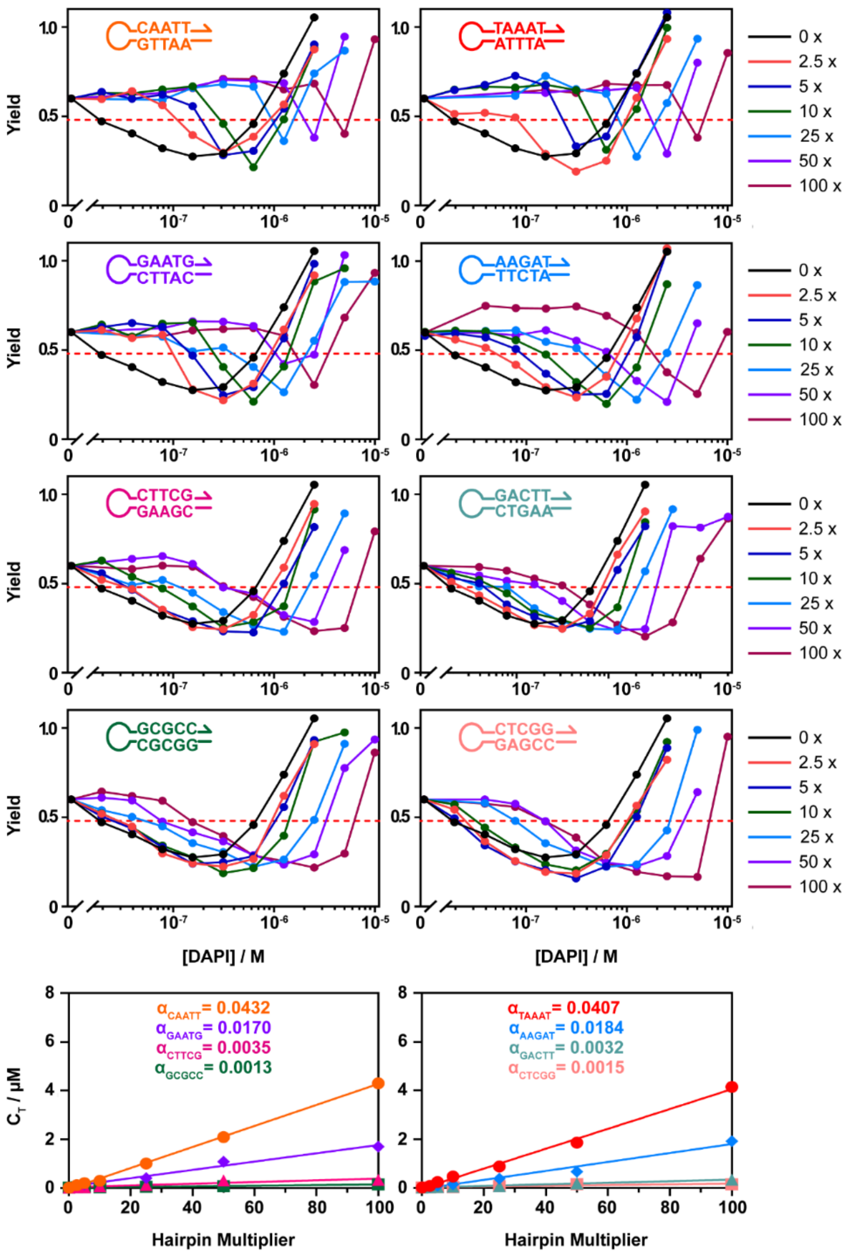

Supplementary Figure 30 | Determining sequence selectivity of DAPI. Eight types of sink probes containing different 5 bp stem sequences were used to interrogate the BIND reaction for DAPI. The shifts of BIND curves were quantitatively analyzed to obtain selectivity factor α. A general trend of sequence preference was determined to be five ≈ four > three > two bp AT binding site for DAPI.

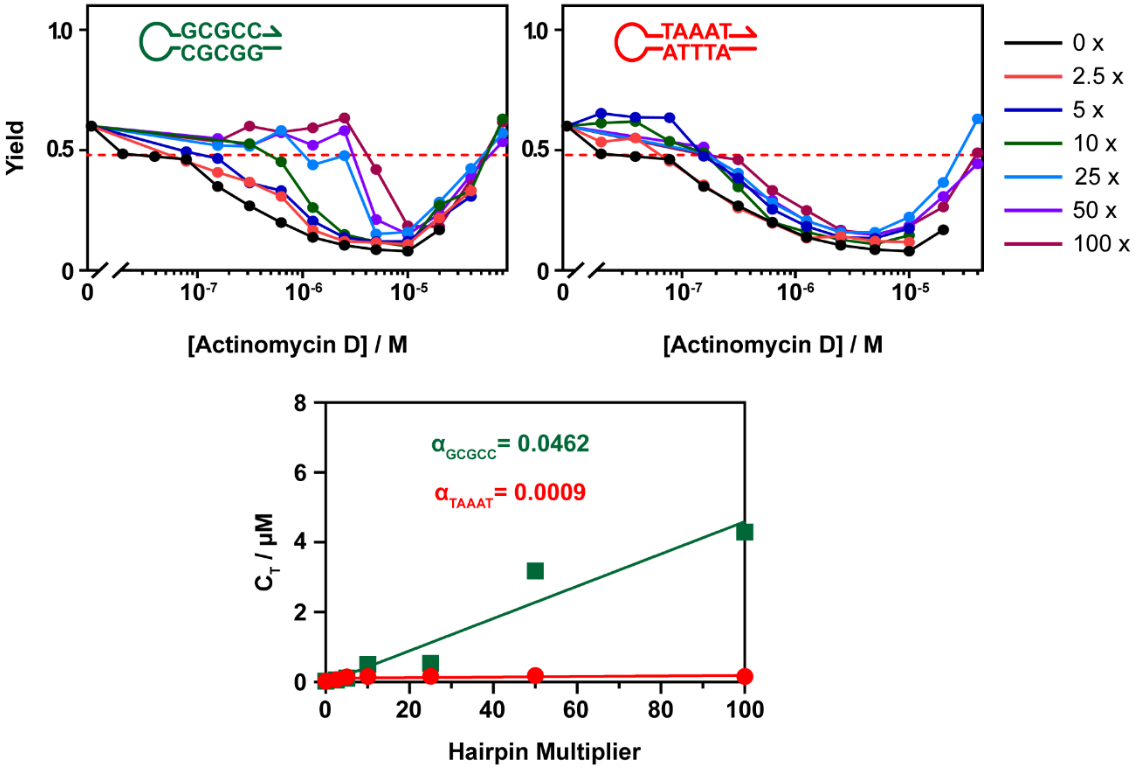

Supplementary Figure 31 | Determining sequence selectivity of Actinomycin D using GC and AT-rich sink probes. BIND reactions were performed in the presence of varying concentrations of sink probes and the shifts in BIND curves were plotted to determine the selectivity factor α through a linear fitting.

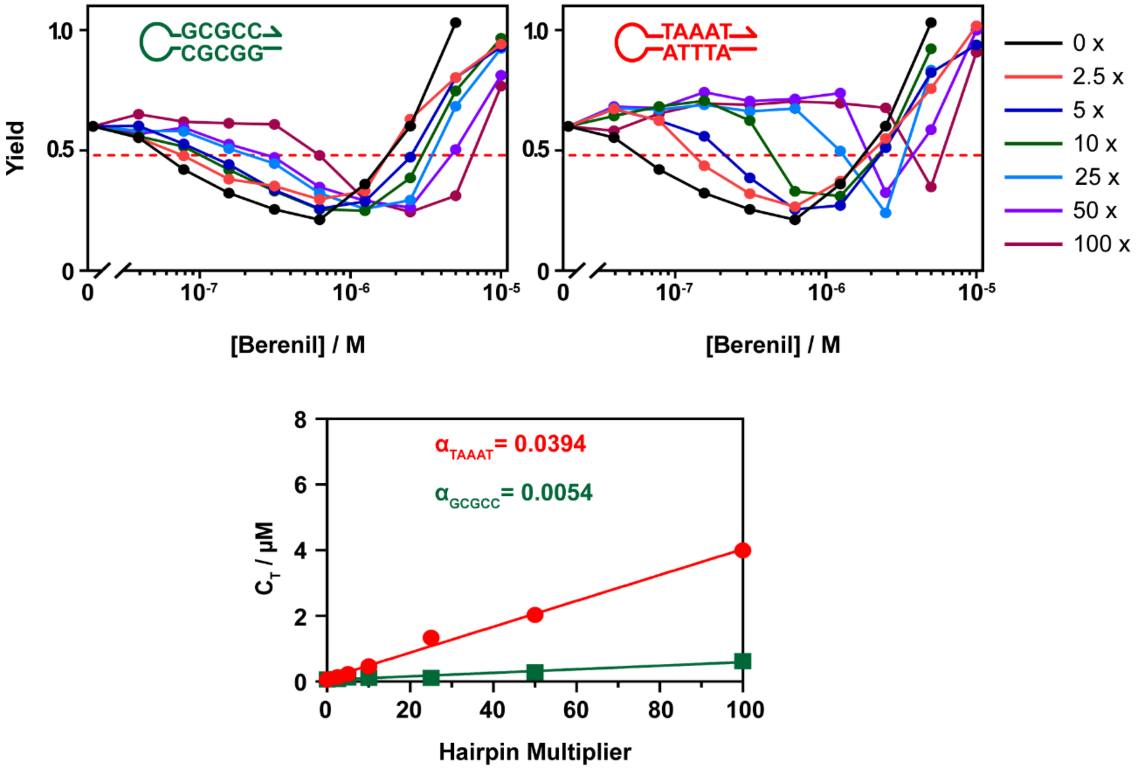

Supplementary Figure 32 | Determining sequence selectivity of Berenil using GC and AT-rich sink probes. BIND reactions were performed in the presence of varying concentrations of sink probes and the shifts in BIND curves were plotted to determine the selectivity factor α through a linear fitting.

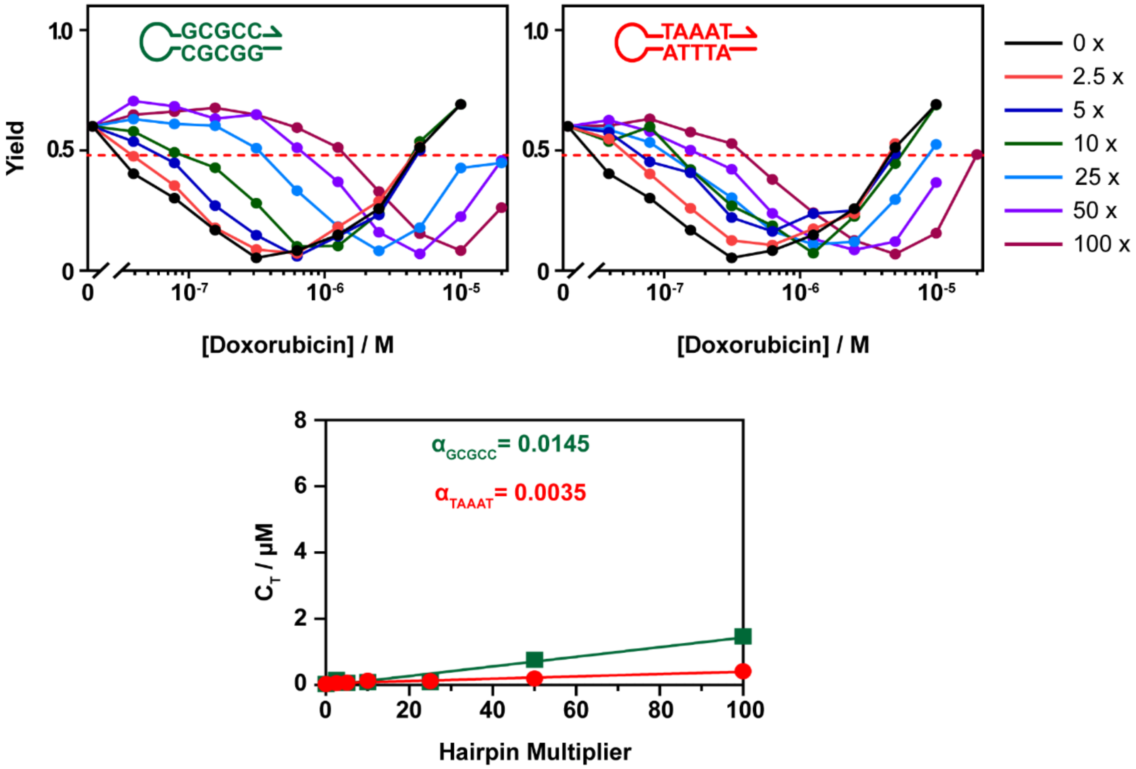

Supplementary Figure 33 | Determining sequence selectivity of Doxorubicin using GC and AT-rich sink probes. BIND reactions were performed in the presence of varying concentrations of sink probes and the shifts in BIND curves were plotted to determine the selectivity factor α through a linear fitting.

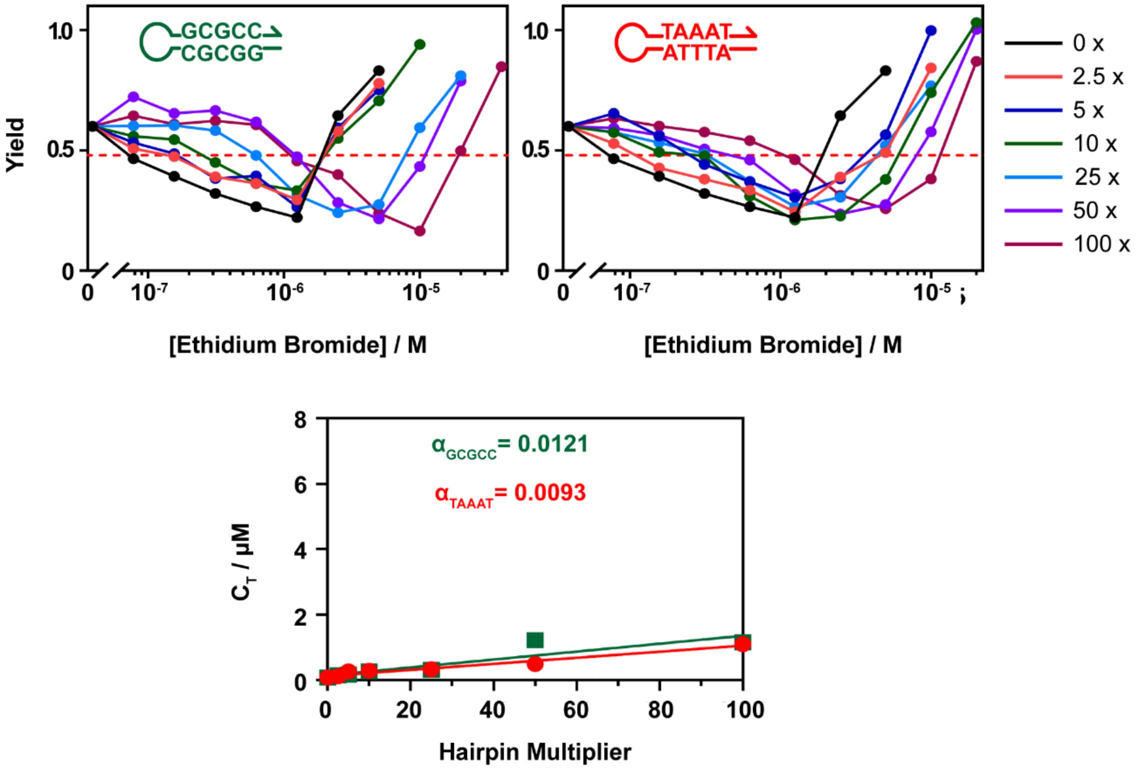

Supplementary Figure 34 | Determining sequence selectivity of Ethidium Bromide using GC and AT-rich sink probes. BIND reactions were performed in the presence of varying concentrations of sink probes and the shifts in BIND curves were plotted to determine the selectivity factor α through a linear fitting.

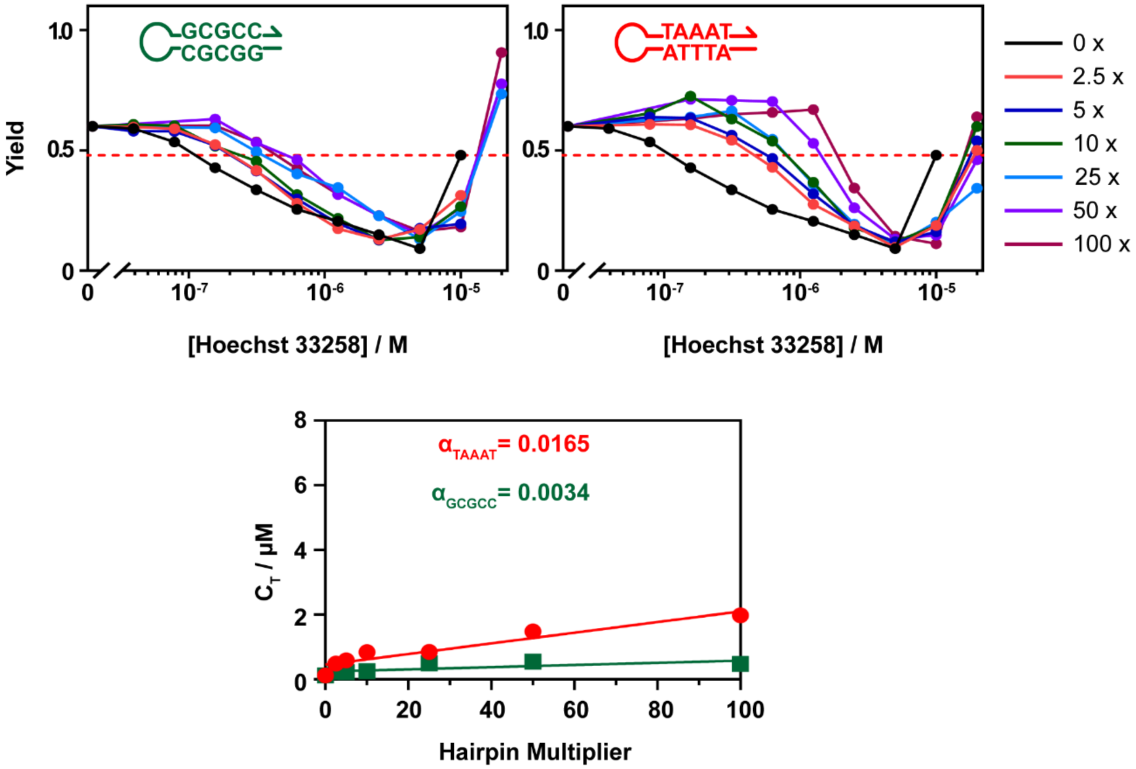

Supplementary Figure 35 | Determining sequence selectivity of Hoechst 33258 using GC and AT-rich sink probes. BIND reactions were performed in the presence of varying concentrations of sink probes and the shifts in BIND curves were plotted to determine the selectivity factor α through a linear fitting.

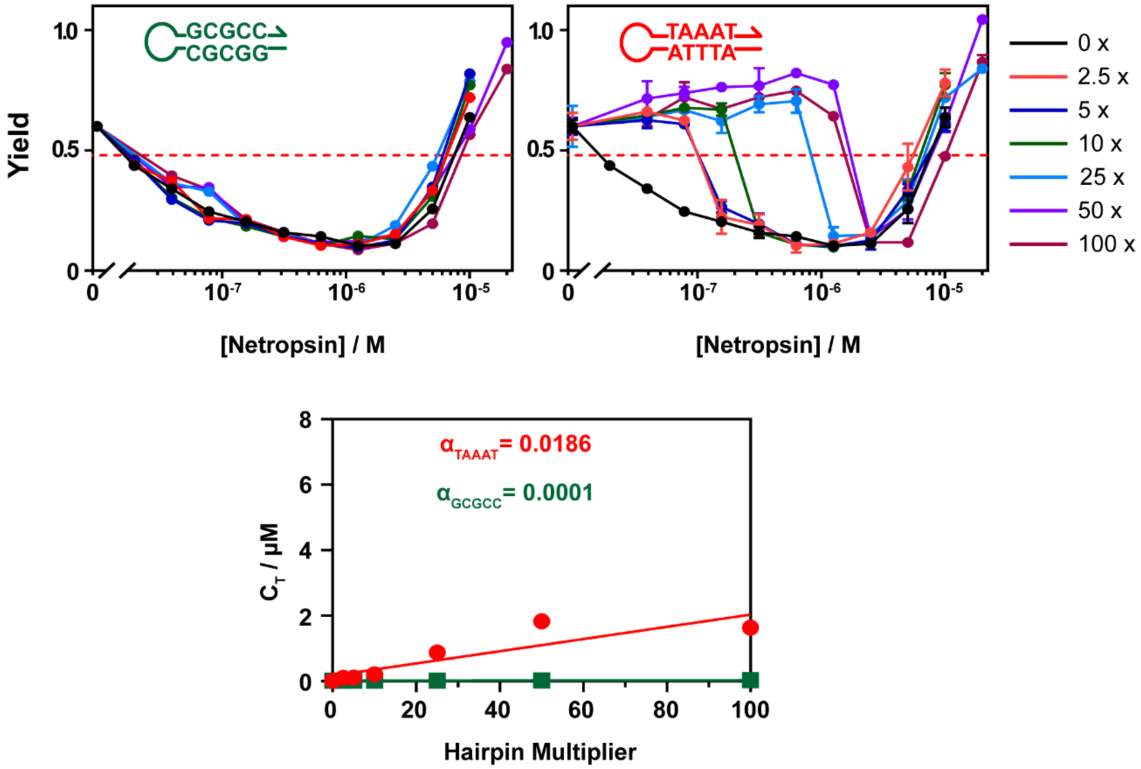

Supplementary Figure 36 | Determining sequence selectivity of Netropsin using GC and AT-rich sink probes. BIND reactions were performed in the presence of varying concentrations of sink probes and the shifts in BIND curves were plotted to determine the selectivity factor α through a linear fitting. Each error bar represents one standard deviation from triplicate analyses (n = 3).

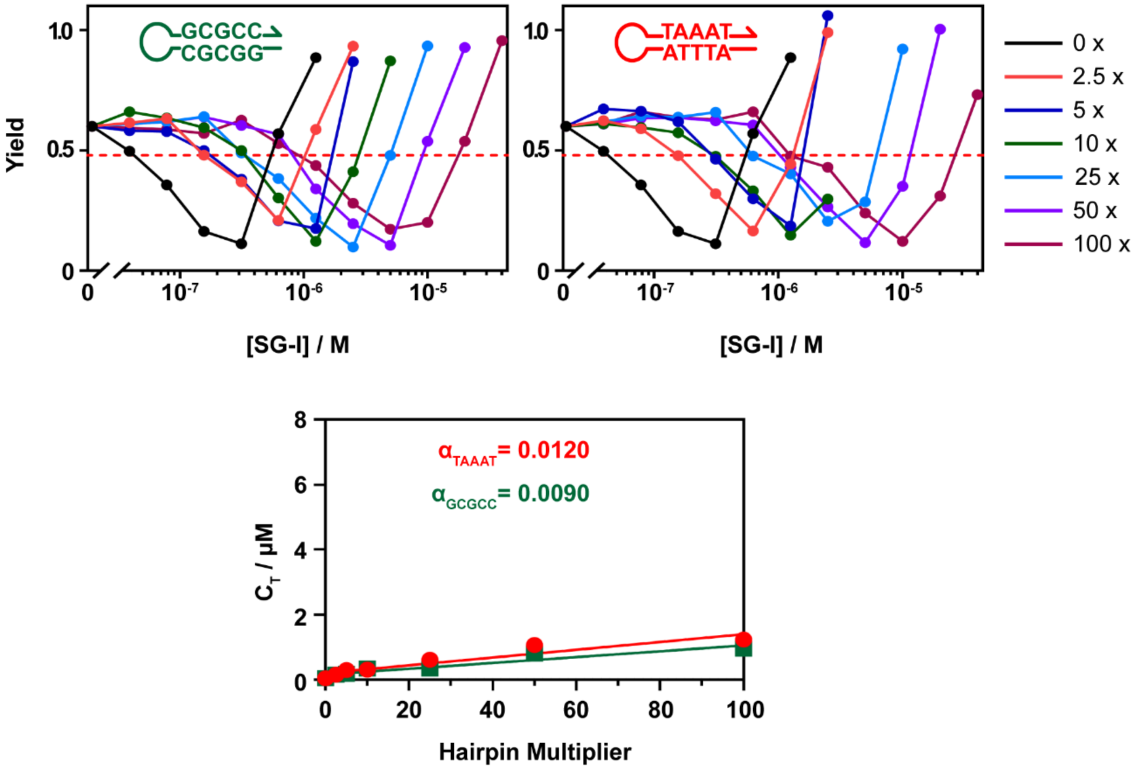

Supplementary Figure 37 | Determining sequence selectivity of SG-I using GC and AT-rich sink probes. BIND reactions were performed in the presence of varying concentrations of sink probes and the shifts in BIND curves were plotted to determine the selectivity factor α through a linear fitting.

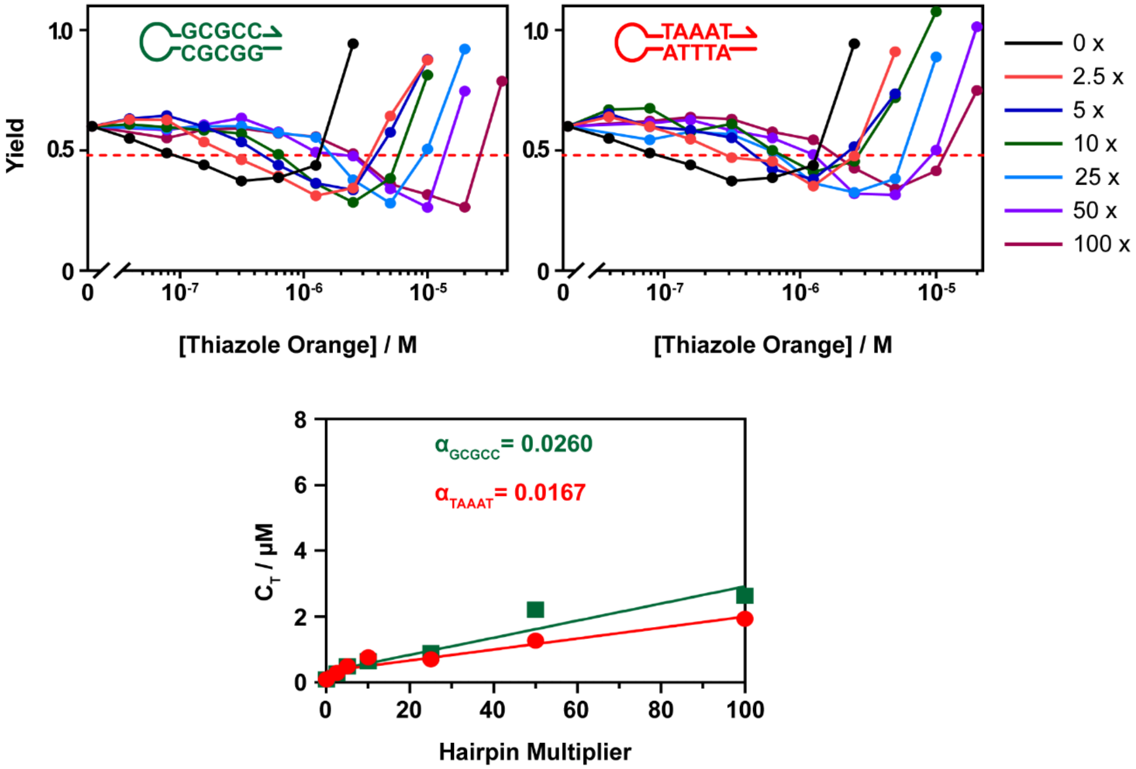

Supplementary Figure 38 | Determining sequence selectivity of Thiazole Orange using GC and AT-rich sink probes. BIND reactions were performed in the presence of varying concentrations of sink probes and the shifts in BIND curves were plotted to determine the selectivity factor α through a linear fitting.

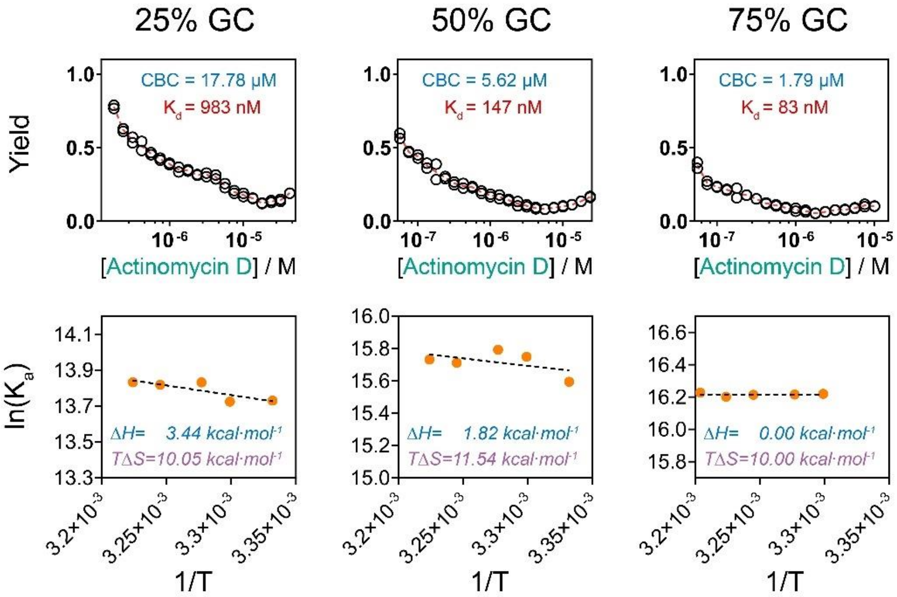

Supplementary Figure 39 | Impact of GC content on BIND. For a GC-selective binder Actinomycin D, the initial fluorescence in the absence of binder decreased as the GC content of duplex increased. This is also to be expected as the CP duplex becomes more stable with higher GC content. Both CBC and K_d values shifted to lower concentrations as we increased the GC content of the duplex. This observation was consistent with the high GC selectivity of Actinomycin D. Thermodynamically, changing the GC content exerts the impact on the binding enthalpy which changed from 3.44 kcal/mol at 25% GC content to 0 kcal/mol at 75% GC content.

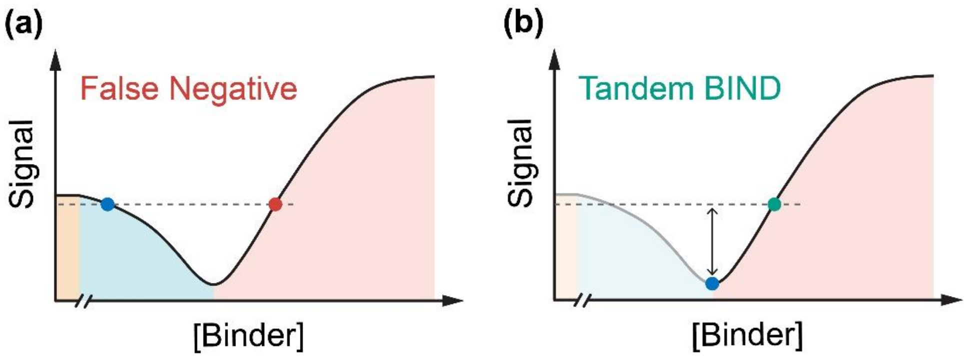

Supplementary Figure 40 | Schematic illustration of the necessity of tandem BIND. **(a)** Schematic illustration of a scenario where a false negative signal was generated when only using BIND for the HTS screening. (**b**) Schematic illustration of how tandem BIND could help address the false negative issue. It is necessary to use a tandem BIND assay because BIND features a hyperbolic curve. If BIND was directly used to perform high throughput screening, the same detection signal may correspond to a negative screening result at the S_N1 domain but may also correspond to a positive result at the S_N2 domain (**a**). To avoid this confusion, the tandem BIND was designed, so that there was a low fluorescence signal at the CBC of the known binder. Upon screening, any fluorescence increase corresponds to the binding of the second binder and returns a positive screening result.

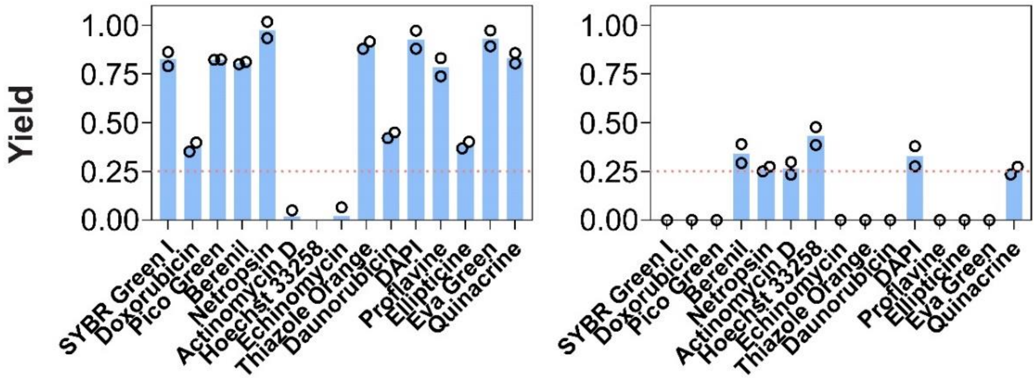

Supplementary Figure 41 | Comparing BIND and FID for re-screening 15 known DNA binders. A total of 15 successfully characterized small DNA binders (except for EtBr) were rescreened by both BIND (**a**) and FID (**b**) HTS assays. Technical replicates (n = 2) were used to ensure the reproducibility of the test.

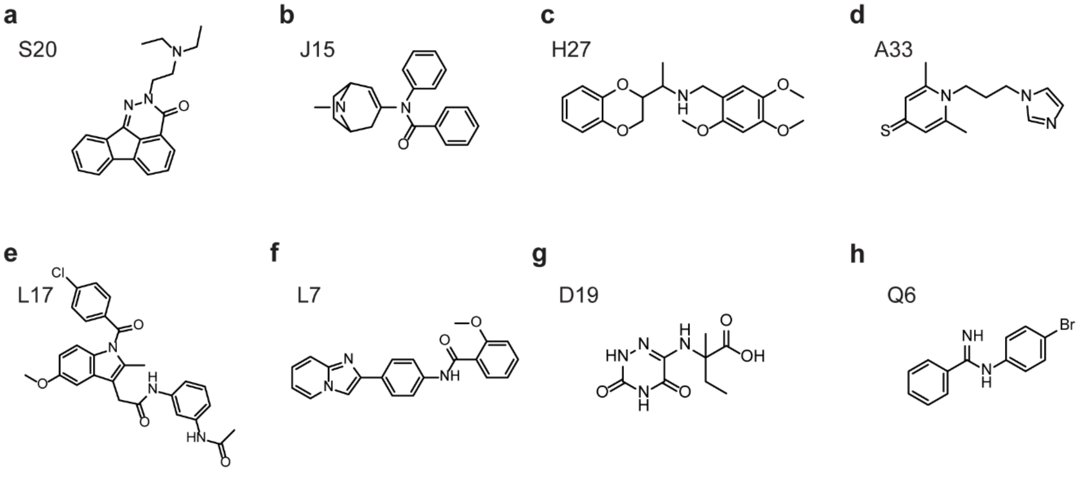

Supplementary Figure 42 | A list of eight positive hits achieved using tandem BIND HTS assay. The eight positive hits were ranked according to the yields in the BIND assay. (**a-h**) Chemical structures of 8 positive hits discovered using the tandem BIND assay with affinities ranked from high to low.

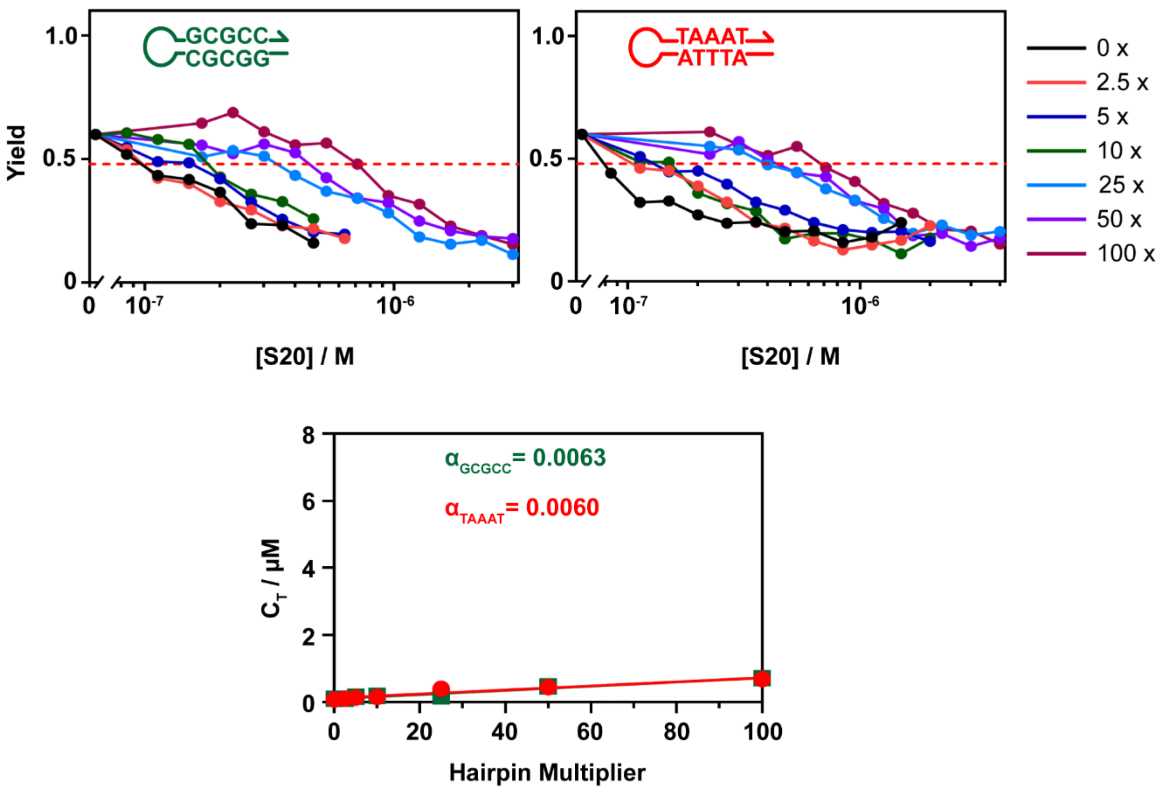

Supplementary Figure 43 | Determining sequence selectivity of the newly discovered DNA binder S20 using GC and AT-rich sink probes. BIND reactions were performed in the presence of varying concentrations of sink probes and the shifts in BIND curves were plotted to determine the selectivity factor α through a linear fitting.

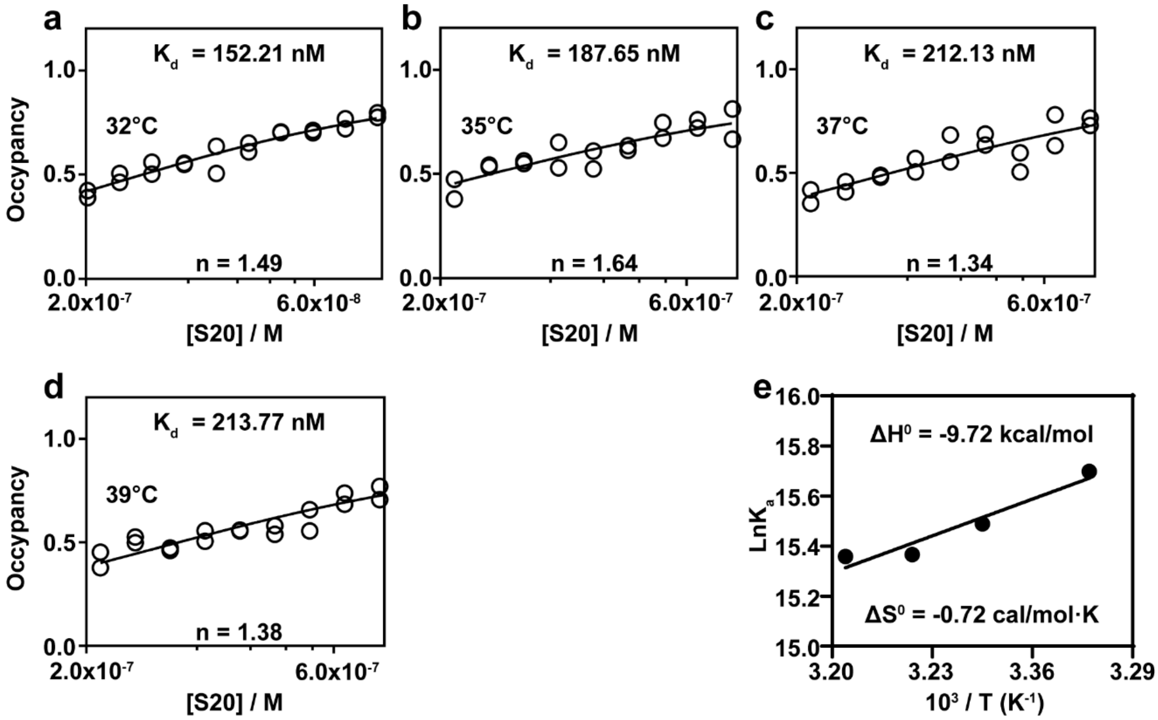

Supplementary Figure 44 | Profiling the binding thermodynamics of the newly discovered DNA binder S20 using BIND. (a-d) BIND-based determination of dissociation constants at 32 ºC (**a**), 35 ºC (**b**), 37 ºC (**c**), and 39 ºC (**d**). **(e)** Determination of △H and △S using linear fitting against Van't Hoff equation. Binding curves established using the S_N1 region of BIND was used to determine the K_d at varying temperatures. Van't Hoff plot was then established to determine the ΔHº and ΔSº.

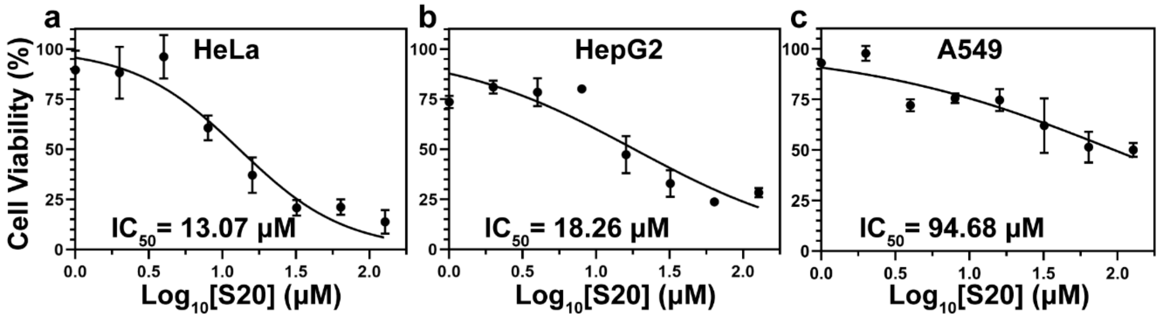

Supplementary Figure 45 | Cytotoxicity study (MTT assay) of S20 with three neoplastic cell lines. The growth inhibition was researched for S20 against 3 cancerous cell lines, a significant growth inhibition was observed among all three malignancies (**a-c**). The 50% inhibition concentration (IC_{50}) was 13.07 μM, 18.26 μM and 94.68 μM for cancerous cell line Hela, HepG2 and A549 respectively. Each error bar represents one standard deviation from triplicate MTT tests (n = 3).

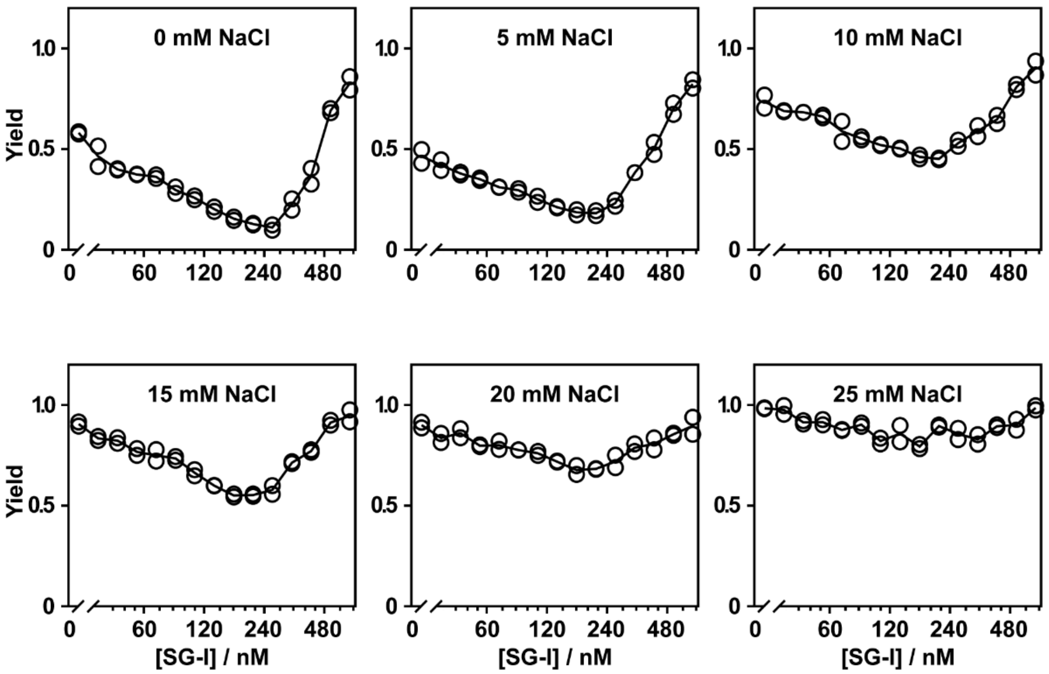

Supplementary Figure 46 | BIND profiles for SG-I in the absence NaCl or the presence of varying NaCl concentrations. BIND profiles were attenuated by increasing concentrations of NaCl and was disappeared when NaCl concentration was raised above 25 mM.

S4. Supplementary Tables

Supplementary Table 1. DNA sequences and modifications.

DNA Names		Sequences
**BIND probes**	**C**	5’-Cy5-AGGTTGGTGAGTGATTGGAGGTT-3’
	**P**	5’-AATCACTCACCAACCT- Iowa Black FQ-3’
	**I**	5’-AACCT CCAATCACTCACCAA CCT-3’
**Sink Probes**	**5’-TAAAT-3’**	5’-CGATTTACAAAAAGTAAATCG-3’
	**5’-CAATT-3’**	5’-CGAATTGCAAAAAGCAATTCG-3’
	**5’-GAATG-3’**	5’-CGCATTCCAAAAAGGAATGCG-3’
	**5’-AAGAT-3’**	5’-CGATCTTCAAAAAG AAGATCG-3’
	**5’-GACTT-3’**	5’-CGAAGTCCAAAAAGGACTTCG-3’
	**5’-CTTCG-3’**	5’-CGCGAAGCAAAAAGCTTCGCG-3’
	**5’-CTCGG-3’**	5’-CGCCGAGCAAAAAGCTCGGCG-3’
	**5’-GCGCC-3’**	5’-CGCGCGGCAAAAAGCCGCGCG-3’
**Difference GC%**	**C (75% GC)**	5’-Cy5-GGGCTACGCGATGCGCGGAGGTT-3’
	**P (75% GC)**	5’-GCGCATCGCGTAGCCC-Iowa Black FQ-3’
	**I (75% GC)**	5’-AACCTCCGCGCATCGCGTAGCCC-3’
	**C (25% GC)**	5’-Cy5- AATTCGATTAGCAATTGGAGGTT-3’
	**P (25% GC)**	5’- AATTGCTAATCGAATT-Iowa Black FQ-3’
	**I (25% GC)**	5’- AACCTCCAATTGCTAATCGAATT-3’
**Different length of CP**	**C (28nt)**	5’-Cy5- ACAAGAGGTTGGTGAGTGATTGGAGGTT-3’
	**P (21nt)**	5’- AATCACTCACCAACCTCTTGT-Iowa Black FQ-3’
	**I (28nt)**	5’- AACCTCCAATCACTCACCAACCTCTTGT-3’
	**C (18nt)**	5’-Cy5- GGTGAGTGATTGGAGGTT-3’
	**P (11nt)**	5’- AATCACTCACC-Iowa Black FQ-3’
	**I (18nt)**	5’- AACCTCCAATCACTCACC-3’

Supplementary Table 2. Comparison of dissociation constants and binding site sizes measured using BIND against reported values.

Binder	K_d / nM (this work)	Binding Site Size (this work)	Reported K_d / nM	Reported Binding Site Size
Actinomycin D	147.1	2.1	125.0[2]	2.5[3]
Echinomycin	246.5	5.6	322.0[4]	5.6[4]
Ellipticine	220.7	2.8	333.3[5]	4.4[6]
DAPP	N/A	N/A	~50000.0[7]	N/A
Quinacrine	0.4	4.8	0.5[8]	5.0[9]
DAPI	1.1	4.4	1.8[10]	4.0[11]
Pico Green	2.5	5.1	5.0[12]	3.8[12]
Netropsin	5.3	5.5	5.0[13]	5.9[14]
SG-`I	7.1	2.6	3.1[15]	3.1[15]
Berenil	17.5	2.2	13.7[16]	1.7[16]
Doxorubicin	20.3	2.9	33.8[17]	3.0[18]
TO	25.6	3.5	66.6[19]	3.1[19]
Daunorubicin	27.0	3.3	86.2[17]	3.2[18]
Eva Green	56.4	3.2	344.8[20]	4.0[20]
Proflavine	31.6	2.2	66.7[21]	2.1[22]
EtBr	67.9	2.4	112.0[23]	2.2[24]
$RuPhen_3Cl_2$	N/A	N/A	113636.4[25]	3.0[25]
Crystal Violate	N/A	N/A	6666.7[26]	4.5[26]
Hoechst 33258	147.9	2.7	204.1[27]	3.0[28]
Thioflavin T	N/A	N/A	14000.0[29]	N/A

Supplementary Table 3. Comparison of enthalpic and entropic contributions measured using BIND against reported values.

Binder	ΔH^0 / ΔG^0	$-T\Delta S^0$ / ΔG^0	ΔH^0 / ΔG^0 reported	$-T\Delta S^0$ / ΔG^0 reported
Actinomycin D	-19%	119%	-16%[30]	116%
Echinomycin	-12%	112%	0%[31]	100%
Ellipticine	49%	51%	39%[32]	61%
DAPP	N/A	N/A	N/A	N/A
Quinacrine	71%	29%	68%[33]	32%
DAPI	6%	94%	33%[34]	67%
Pico Green	-4%	114%	N/A	N/A
Netropsin	83%	17%	76%[14]	24%
SG-I	3%	97%	N/A	N/A
Berenil	75%	25%	74%[35]	26%
Doxorubicin	80%	20%	83%[36]	17%
TO	133%	-33%	N/A	N/A
Daunorubicin	95%	5%	84%[37]	16%
Eva Green	N/A	N/A	N/A	N/A
Proflavine	72%	28%	79%[38]	21%
EtBr	119%	-19%	131%[39]	-31%
$RuPhen_3Cl_2$	N/A	N/A	N/A	N/A
Crystal Violate	N/A	N/A	N/A	N/A
Hoechst 33258	-20%	120%	-37%[40]	137%
Thioflavin T	N/A	N/A	N/A	N/A

Supplementary References

1. Glass, L.S., Bapat, A., Kelley, M.R., Georgiadis, M.M. & Long, E.C. Semi-automated high-throughput fluorescent intercalator displacement-based discovery of cytotoxic DNA binding agents from a large compound library. *Bioorg Med Chem Lett* **20**, 1685-1688 (2010).
2. Chen, F.-M., Sha, F., Chin, K.-H. & Chou, S.-H. The nature of actinomycin D binding to d(AACCAXYG) sequence motifs. *Nucleic acids research* **32**, 271-277 (2004).
3. Lohani, N., Singh, H.N. & Rajeswari, M.R. Assessment of binding properties of Actinomycin-D to 21nt DNA segment of hmgb1 gene promoter using spectroscopic and calorimetric techniques. *Journal of biomolecular structure & dynamics* **36**, 504-511 (2018).
4. Wakelin, S.P. & Waring, M.J. The binding of echinomycin to deoxyribonucleic acid. *Biochemical journal* **157**, 721-740 (1976).
5. Ghosh, S., Kar, A., Chowdhury, S. & Dasgupta, D. Ellipticine Binds to a Human Telomere Sequence: An Additional Mode of Action as a Putative Anticancer Agent? *Biochemistry (Easton)* **52**, 4127-4137 (2013).
6. Dodin, G., Schwaller, M.A., Aubard, J. & Paoletti, C. Binding of ellipticine base and ellipticinium cation of calf-thymus DNA: a thermodynamic and kinetic study. *European journal of biochemistry* **176**, 371-376 (1988).
7. Kubař, T., Hanus, M., Ryjáček, F. & Hobza, P. Binding of Cationic and Neutral Phenanthridine Intercalators to a DNA Oligomer Is Controlled by Dispersion Energy: Quantum Chemical Calculations and Molecular Mechanics Simulations. *Chemistry : a European journal* **12**, 280-290 (2005).
8. Wilson, W.D. & Lopp, I.G. Analysis of cooperativity and ion effects in the interaction of quinacrine with DNA. *Biopolymers* **18**, 3025-3041 (1979).
9. Sumner, A.T. Mechanisms of quinacrine binding and fluorescence in nuclei and chromosomes. *Histochemistry (Berlin)* **84**, 566-574 (1986).
10. Breusegem, S.Y., Clegg, R.M. & Loontiens, F.G. Base-sequence specificity of Hoechst 33258 and DAPI binding to five (A/T) 4 DNA sites with kinetic evidence for more than one high-affinity Hoechst 33258-AATT complex. *Journal of molecular biology* **315**, 1049-1061 (2002).
11. Trotta, E., Del Grosso, N., Erba, M. & Paci, M. The ATT strand of AAT.ATT trinucleotide repeats adopts stable hairpin structures induced by minor groove binding ligands. *Biochemistry (Easton)* **39**, 6799-6808 (2000).
12. Dragan, A.I. et al. Characterization of PicoGreen Interaction with dsDNA and the Origin of Its Fluorescence Enhancement upon Binding. *Biophysical journal* **99**, 3010-3019 (2010).
13. Premvardhan, L. & Maurizot, J.-C. Netropsin binding in five duplex-dimer DNA constructs as a function of size and distance between binding sites: circular dichroism and absorption spectroscopy. *European biophysics journal* **39**, 781-787 (2010).
14. Vardevanyan, P.O., Parsadanyan, M.A., Antonyan, A.P. & Sahakyan, V.G. Spectroscopic Study of the Binding of Netropsin and Hoechst 33258 to Nucleic Acids. *Journal of applied spectroscopy* **85**, 335-340 (2018).

15. Dragan, A.I. et al. SYBR Green I: Fluorescence Properties and Interaction with DNA. *Journal of fluorescence* **22**, 1189-1199 (2012).
16. Pilch, D.S., Kirolos, M.A., Liu, X., Plum, G.E. & Breslauer, K.J. Berenil [1,3-Bis(4'-amidinophenyl)triazene] Binding to DNA Duplexes and to a RNA Duplex: Evidence for Both Intercalative and Minor Groove Binding Properties. *Biochemistry (Easton)* **34**, 9962-9976 (1995).
17. Chaires, J.B. et al. Parsing the Free Energy of Anthracycline Antibiotic Binding to DNA. *Biochemistry (Easton)* **35**, 2047-2053 (1996).
18. Barcelo, F., Martorell, J., Gavilanes, F. & Gonzalez-Ros, J.M. Equilibrium binding of daunomycin and adriamycin to calf thymus DNA: Temperature and ionic strength dependence of thermodynamic parameters. *Biochemical pharmacology* **37**, 2133-2138 (1988).
19. Boger, D.L. & Tse, W.C. Thiazole orange as the fluorescent intercalator in a high resolution fid assay for determining DNA binding affinity and sequence selectivity of small molecules. *Bioorganic & medicinal chemistry* **9**, 2511-2518 (2001).
20. Shoute, L.C.T. & Loppnow, G.R. Characterization of the binding interactions between EvaGreen dye and dsDNA. *Physical chemistry chemical physics : PCCP* **20**, 4772-4780 (2018).
21. MoradpourHafshejani, S., Hedley, J.H., Haigh, A.O., Pike, A.R. & Tuite, E.M. Synthesis and binding of proflavine diazides as functional intercalators for directed assembly on DNA. *RSC advances* **3**, 18164 (2013).
22. Aslanoglu, M. Electrochemical and Spectroscopic Studies of the Interaction of Proflavine with DNA. *Analytical sciences* **22**, 439-443 (2006).
23. Hinton, D.M. & Bode, V.C. Ethidium binding affinity of circular lambda deoxyribonucleic acid determined fluorometrically. *The Journal of biological chemistry* **250**, 1061-1070 (1975).
24. Vardevanyan, P.O. et al. The influence of GC/AT composition on intercalating and semi-intercalating binding of ethidium bromide to DNA. *Journal of the Brazilian Chemical Society* **23**, 2016-2020 (2012).
25. Mihailovic, A. et al. Exploring the Interaction of Ruthenium(II) Polypyridyl Complexes with DNA Using Single-Molecule Techniques. *Langmuir* **22**, 4699-4709 (2006).
26. Wakelin, L.P.G., Adams, A., Hunter, C. & Waring, M.J. Interaction of crystal violet with nucleic acids. *Biochemistry (Easton)* **20**, 5779-5787 (1981).
27. Guan, Y., Shi, R., Li, X., Zhao, M. & Li, Y. Multiple Binding Modes for Dicationic Hoechst 33258 to DNA. *The journal of physical chemistry. B* **111**, 7336-7344 (2007).
28. Vardevanyan, P.O. et al. Influence of Ionic Strength on Hoechst 33258 Binding with DNA. *Journal of biomolecular structure & dynamics* **25**, 641-646 (2008).
29. Liu, S., Peng, P., Wang, H., Shi, L. & Li, T. Thioflavin T binds dimeric parallel-stranded GA-containing non-G-quadruplex DNAs: a general approach to lighting up double-stranded scaffolds. *Nucleic acids research* **45**, 12080-12089 (2017).
30. Galo, A.L. et al. The Influence of Solutes on the Enthalpy/Entropy Change of the Actinomycin D Binding to DNA: Hydration, Energy Compensation and Long-Range Deformation on DNA. *The journal of physical chemistry. B* **115**, 8883-8890 (2011).

31. L. P. G, W. & M. J, W. The Unwinding of Circular Deoxyribonucleic Acid by Phenanthridinium Drugs: Structure-Activity Relations for the Intercalation Reaction. *Molecular pharmacology* **10**, 544-561 (1974).
32. Schwaller, M.A., Dodin, G. & Aubard, J. Thermodynamics of drug–DNA interactions: Entropy-driven intercalation and enthalpy-driven outside binding in the ellipticine series. *Biopolymers* **31**, 519-527 (1991).
33. Hossain, M., Giri, P. & Kumar, G.S. DNA intercalation by quinacrine and methylene blue: a comparative binding and thermodynamic characterization study. *DNA and cell biology* **27**, 81-90 (2008).
34. Daniel, S.P. et al. A Terbenzimidazole that Preferentially Binds and Conformationally Alters Structurally Distinct DNA Duplex Domains: A Potential Mechanism for Topoisomerase I Poisoning. *Proceedings of the National Academy of Sciences - PNAS* **94**, 13565-13570 (1997).
35. Schmitz, H.U. & Hübner, W. A thermodynamic and spectroscopic study on the binding of berenil to poly d(AT) and to poly (dA) · poly (dT). *Biophysical chemistry* **48**, 61-74 (1993).
36. Schneider, Y.J., Baurain, R., Zenebergh, A. & Trouet, A. DNA-binding parameters of daunorubicin and doxorubicin in the conditions used for studying the interaction of anthracycline-DNA complexes with cells in vitro. *Cancer chemotherapy and pharmacology* **2**, 7-10 (1979).
37. Remeta, D.P., Mudd, C.P., Berger, R.L. & Breslauer, K.J. Thermodynamic characterization of daunomycin-DNA interactions: microcalorimetric measurements of daunomycin-DNA binding enthalpies. *Biochemistry (Easton)* **30**, 9799-9809 (1991).
38. Quadrifoglio, F., Crescenzi, V. & Giancotti, V. Calorimetry of DNA-dye interactions in aqueous solution: I. Proflavine and ethidium bromide. *Biophysical chemistry* **1**, 319-324 (1974).
39. Chou, W.Y., Marky, L.A., Zaunczkowski, D. & Breslauer, K.J. The Thermodynamics of Drug-DNA Interactions: Ethidium Bromide and Propidium Iodide. *Journal of biomolecular structure & dynamics* **5**, 345-359 (1987).
40. Haq, I., Ladbury, J.E., Chowdhry, B.Z., Jenkins, T.C. & Chaires, J.B. Specific binding of hoechst 33258 to the d(CGCAAATTTGCG) 2 duplex: calorimetric and spectroscopic studies. *Journal of molecular biology* **271**, 244-257 (1997).
